# Effectiveness and implementation of a multi-faceted intervention to facilitate adoption of asthma self-management practices in Peruvian children and adolescents: a hybrid type 2 individually randomized controlled trial

**DOI:** 10.3389/fpubh.2025.1710746

**Published:** 2026-01-13

**Authors:** Alejandro Zevallos-Morales, Elisa Romani-Huacani, Kevin J. Psoter, Oscar Flores-Flores, David Reif, Trishul Siddharthan, Mariona Portell, Maria Teresa Anguera, Lindsay J. Underhill, Suzanne L. Pollard

**Affiliations:** 1 Universidad de San Martin de Porres, Facultad de Medicina Humana, Centro de Investigación del Envejecimiento (CIEN), Lima, Peru; 2 Department of Psychobiology and Methodology of Health Sciences, Universitat Autònoma de Barcelona, Cerdanyola del Vallès, Spain; 3 Universidad Tecnológica del Perú, Lima, Peru; 4 Division of General Pediatrics, Johns Hopkins University School of Medicine, Baltimore, MD, United States; 5 Department of International Health, Bloomberg School of Public Health, Johns Hopkins University, Baltimore, MD, United States; 6 Division of Pulmonary and Critical Care, Johns Hopkins University School of Medicine, Baltimore, MD, United States; 7 Division of Pulmonary, Critical Care and Sleep Medicine, University of Miami, Miami, FL, United States; 8 Faculty of Psychology, Institute of Neurosciences, University of Barcelona, Barcelona, Spain; 9 Cardiovascular Division, Washington University in St. Louis School of Medicine, St. Louis, MO, United States

**Keywords:** asthma self-management, asthma education, clinical trial, pediatric asthma, quality of life

## Abstract

**Background:**

Peru has one of the highest burdens of asthma in the world, as well as large gaps in access to evidence-based treatments. Studies often overlook the ways in which the research context and data collection activities interact and influence the experience of a research participant, their perception of an intervention, and, by extension, study outcomes.

**Methods:**

We conducted an individually randomized type 2 hybrid-implementation trial to evaluate the implementation and effectiveness of a locally adapted, multi-faceted intervention package to improve adherence to self-management practices. We enrolled 110 children with physician-diagnosed asthma living in nine urban districts Lima, Peru, and followed them monthly for 6 months. 101 children completed the study. Participants in the intervention group received case management from a designated nurse manager, who provided ongoing educational, social, and self-management support in the form of follow-up home visits and phone-based communication. Long-term inhaler therapy was provided free of charge to both the intervention and control groups. We measured clinical effectiveness and implementation outcomes, guided by the Reach, Effectiveness, Adoption, Implementation, Maintenance framework.

**Results:**

Overall, both arms of the study saw improvements in asthma control, adherence, child- and caregiver- quality of life, and caregiver depressive symptoms, with the greatest improvement occurring between baseline and 1 month follow-up. However, there were no statistical differences in these outcomes between intervention and control arms at 6 months. Most caregivers in both arms perceived an improvement in asthma control and wellbeing. Caregivers and nurse managers found the intervention acceptable. Our results suggest that data collection activities involving regular check-ins regarding asthma symptoms and quality of life, as well as medication provision, may have contributed to improvements in clinical outcomes in both arms and partially explain the null results between arms.

**Discussion:**

Our study highlights the need for structural solutions, particularly around medication availability and affordability, for improving asthma management in these contexts. Our study design and analytical approach allowed us to identify underlying mechanisms that contributed to improvements in both study arms, despite the absence of a statistically significant effect in the primary outcome analysis. Work clearly accessible to a broad readership.

**Clinical trial registration:**

ClinicalTrials.gov, https://clinicaltrials.gov/study/NCT03986177.

## 1 Introduction

Despite the existence of several evidence-based biomedical and behavioral interventions for asthma management, including effective medications and supported self-management practices, children in in low- and middle-income countries (LMICs) experience disproportionally high morbidity and mortality due to asthma (1, 2). These disparities are largely driven by significant barriers to care, including under-resourced healthcare systems, a shortage of trained providers, complex referral and medication procurement systems, and low availability and financial access to preventive medications (3), among many others. The overall result is poorer health outcomes and lower quality of life for children and caregivers, as well as increased risk for other chronic lung diseases in later life (4).

There is growing recognition of the importance of evaluating implementation processes in behavioral intervention trials, including in asthma intervention trials (5, 6). Still, many studies continue to prioritize clinical effectiveness outcomes at the expense of fully examining the contextual or design-related factors that underlie those results. In null trials especially, evaluating both clinical and implementation outcomes is critical, as null results may not always reflect a “failure” of the intervention, but rather implementation challenges or limitations in the chosen study design or methods. Employing mixed methods approaches within hybrid effectiveness-implementation designs (7) can help address this gap and enhance the utility of study findings. Thus, there is a clear need for studies that employ systematic, mixed methods approaches to address context-specific barriers to evidence-based asthma care in LMIC settings.

The main objective of the Asthma Implementation Research (AIRE) study, a hybrid type 2 effectiveness-implementation individually randomized controlled trial (7), was to evaluate the effectiveness and implementation of a locally adapted intervention package designed to improve adoption of asthma self-management practices among children living in low-resource communities in Lima, Peru, a city with a high burden of asthma and large treatment gap (3, 8). Guided by the Reach, Effectiveness, Adoption, Implementation, Maintenance (RE-AIM) (9), we carried out a mixed methods outcomes analysis embedded within this hybrid trial (7). This approach allowed us to document not only the processes and outcomes that would be relevant in real-world implementation of this intervention in this context, but also to understand the ways in which the research context and data collection activities interact and influence the experience of research participants, their perception of an intervention, and, by extension, study outcomes.

## 2 Methods

### 2.1 Study setting and recruitment

Between January 2019 and March 2020, participants were recruited from a pediatric pulmonary clinic at Cayetano Heredia Hospital located in the district of San Martín de Porres in northern Lima, Peru. This clinic primarily served children and adolescents who had been referred by other providers or through the emergency room for uncontrolled or difficult to manage asthma. Physicians informed individuals who were likely to meet eligibility criteria about the research study. Participants that expressed interest were referred to study personnel on site and then completed a screening questionnaire. Those who met inclusion criteria completed written informed parental consent and written informed assent for children. Inclusion criteria included (1) currently living in any of nine districts in the hospital catchment area; (2) aged 5–17 years; (3) had a prior physician’s diagnosis of asthma; and (4) attended the emergency room or a clinic consultation for asthma at least once in the previous 12 months. Exclusion criteria included: (1) plans to move out of the study community within the next 6 months; (2) co- occurring respiratory or cardiovascular disorders other than asthma; and (3) active tuberculosis or currently taking tuberculosis medications.

### 2.2 Study design

**Figure 1:**
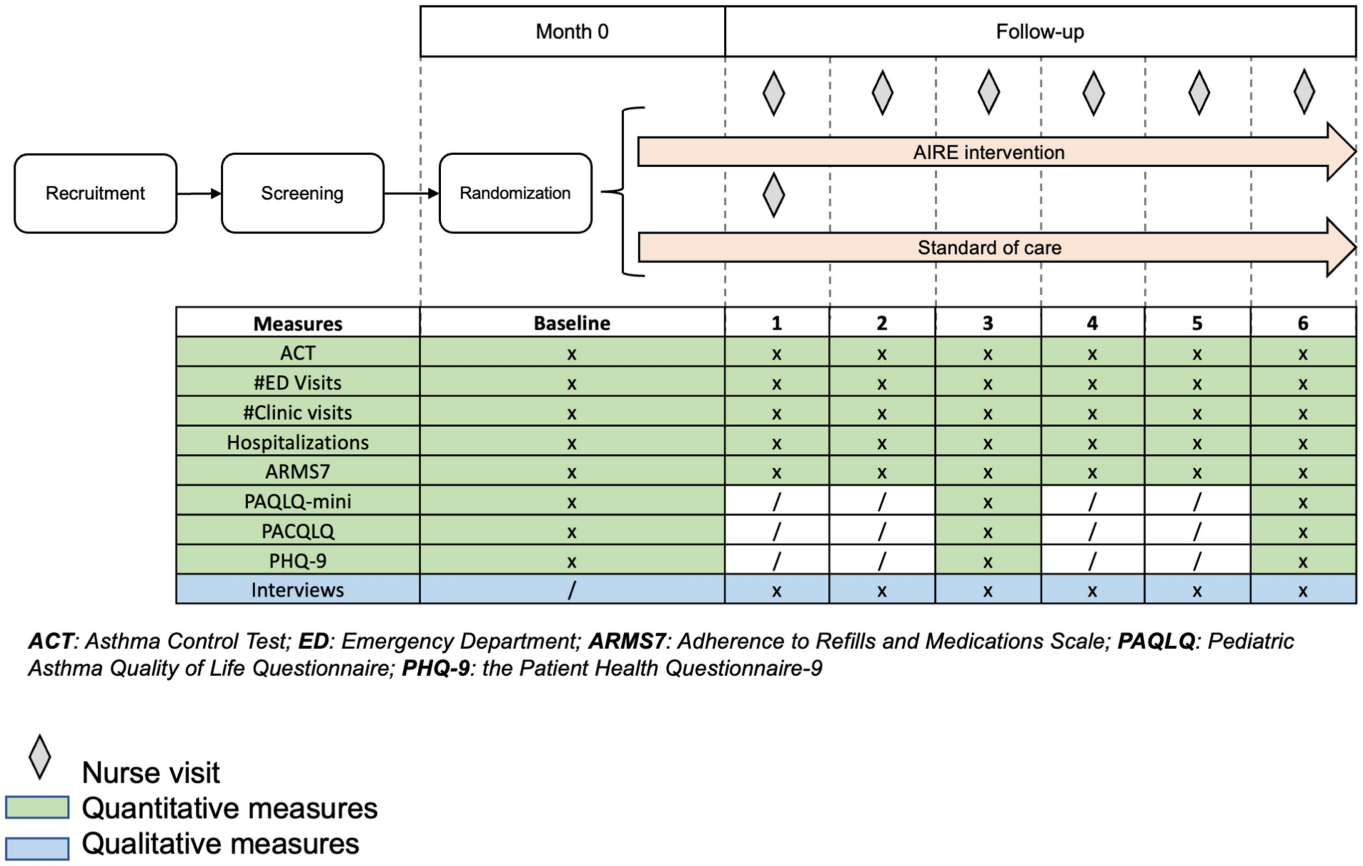
Detailed overview of study timeline, including nurse visits and outcome assessments from data collectors over the course of the study.

The study protocol was previously published^1^ (10). Briefly, the primary study design was an hybrid type 2 individually randomized controlled trial. We aimed to evaluate the implementation and effectiveness of a multi-faceted asthma self-management intervention in a low-resource setting in Lima, Peru. We used a concurrent mixed methods approach, whereby qualitative and quantitative data were collected simultaneously, with integration occurring during the interpretation phase (11). Data collectors followed up with participants monthly for a six-month period. In Figure 1, we provide an overview of the study timeline, including details on nurse visit frequency and the timing of outcome assessments. Participants were randomized 1:1 to either the intervention or control arm using the Sealed Envelope Randomization Service (12). We used block randomization with randomly permuted block sizes of 4, 6, and 8. The principal investigator and data analyst were masked to treatment assignment; masking of field personnel and study participants was not feasible due to the nature of the intervention.

### 2.3 Intervention and control conditions

Intervention development was guided by the Capability, Opportunity, Motivation-Behavior/Behavior Change Wheel framework and is described in detail elsewhere (10). Using prior formative research, we identified and selected key barriers to adoption and sustainment of self-management practices, considering both potential impact and feasibility (13). We then selected behavior change techniques and modes of delivery to target these key barriers and developed a Theory of Change (10).

The intervention group had a designated nurse manager who provided monthly home visits and were available via cell phone and text message throughout the six-month follow-up period. They were recruited from public first-level primary care facilities in Lima and completed a structured one-month training program consisting of weekly sessions led by a general practitioner and a pediatric pulmonologist from the study team. This program included pediatric asthma pathophysiology, guideline-based pharmacologic management, and the content and use of the resources used in the intervention. Nurses were also trained in communication skills for home-based education. A more detailed explanation of the training material can be seen in the pilot paper (10). Nurse managers checked in monthly about symptoms, medication adherence, and any other concerns. They also received a contextually adapted personalized asthma action plan, and an individualized patient-provider communication tool (“Asma Card”). The Asma Card facilitated nurses in providing structured feedback and support on asthma self-management practices as appropriate. Nurses also assisted families in navigating the health system. The intervention also included an education program based on the National Heart, Lung, and Blood Institute (NHLBI) “Respirar es vida / A Breath of Life: Asthma Control for My Child” curriculum that covered asthma pathophysiology, symptom recognition, medication use and inhaler technique, and environmental trigger reduction using interactive learning (e.g., flipcharts, videos, discussion, monitoring and feedback, demonstrations). These concepts were evaluated and reinforced during monthly visits. Intervention participants also received the “Super Niño” booklet that provided interactive, action-oriented educational material in a comic book format (see Supplementary material).

The control group received a one-time basic asthma education visit from a nurse manager for management and prevention of exacerbations. Participants in both arms received access to inhaled corticosteroids as prescribed by their physician, free of charge, for the duration of the follow-up period. Data collectors engaging with participants in both arms were instructed to not provide any direct health advice to participants.

All instruments used in the study were created or adapted to the Peruvian context through an iterative feedback process from local general practitioners, pulmonologists, children with asthma, and their caregivers during the formative pilot phase (10). Discussions with a Community Advisory Board of caregivers of children with asthma in this community informed the content and materials of the intervention. Further, critical information regarding barriers caregivers experience in these settings was provided. The study design, protocol, and data collection activities were all co-led by Dr. Romani, a senior nurse and epidemiologist in the study community with in-depth knowledge of the local context.

### 2.4 Procedures and outcomes

We used the RE-AIM framework (9) to guide selection and analysis of clinical effectiveness and implementation outcomes (Table 1). Because our study had a prescribed set of eligibility criteria, and the intervention was carried out by trained research staff, we de-emphasized reach and adoption (among implementers) and focused on the effectiveness (clinical effectiveness and participant engagement with intervention components), and implementation (acceptability, feasibility, fidelity, dose) domains.

**Table 1:** Summary of reach, effectiveness, adoption, implementation, and effectiveness outcomes evaluated with mixed methods approaches.

Domain	Outcome measure	
Reach	Quantitative	Qualitative
Participant level	Differences between AIRE intervention participants and control population.	
Caregiver	Differences between caregivers from AIRE intervention participants and control population.	In-depth interviews
Effectiveness: effect of AIRE intervention in asthma management at month 6		
Individual	Primary outcome: ACT score	
	Secondary outcomes: Healthcare utilization (Number of emergency visits, physician visits, and hospitalizations)	
	Secondary outcomes: Medication adherence (ARMS7)	
	Secondary outcomes: Participant quality of life (PAQLQ)	
Caregiver	Secondary outcomes: Caregiver quality of life (PACQLQ)	In-depth interviews evaluating the effectiveness of AIRE intervention perceived by caregivers
	Secondary outcomes: Caregiver depressive symptoms (PHQ-9)	
Adoption and implementation		
Participant level	Percentage of participants loss to follow up (per visit basis)	Audio recordings of interactions between nurses and study participants, field notes
Caregiver		In-depth interviews of caregivers, related to the action plan & education
Nurses	Number of visits attempted and conducted	Audio recordings of interactions between nurses and study participants and field notes (Fidelity checklist)
	Number of calls attempted and conducted	In-depth interviews identifying modifications/adaptations of the intervention to the population
Maintenance	Not evaluated	Not evaluated

#### 2.4.1 Clinical effectiveness

The *primary clinical effectiveness* outcome was asthma control at 6 months of follow-up, measured using the Childhood Asthma Control Test (cACT) for ages 5 to 11, or the adolescent Asthma Control Test (aACT) for ages 12 to 17 (14). We assessed ACT score at baseline and at each monthly follow-up visit, for a total of seven measurements. We also evaluated all-cause hospitalizations at monthly visits.

*Medication adherence* was evaluated at baseline and monthly using the Adherence to Refills and Medications Scale (ARMS-7) (15). In children, Disease-specific quality of life (QOL) was assessed using the Pediatric Asthma Quality of Life Questionnaire (PAQLQ)-Mini at baseline, 3, and 6 months (16). We also measured caregiver quality of life using the Pediatric Asthma Caregiver’s Quality of Life Questionnaire (PACQLQ) (17). Depressive symptoms were measured in adult caregivers with the Patient Health Questionnaire-9 (PHQ-9) (18). Finally, we collected information regarding the number of physician consultations.

##### 2.4.1.1 Caregiver perception of effectiveness, intervention engagement, acceptability, feasibility

We carried out in-depth interviews with 53 caregivers in the last month of follow-up (27 in the intervention group, 26 in the control group) and two nurse managers to inform our analysis of perceived effectiveness, engagement with the intervention, acceptability, and feasibility. We also used detailed field notes from nurse managers to inform our understanding of feasibility and identify barriers to implementation and potential future adaptations.

##### 2.4.1.2 Fidelity and dose

We evaluated fidelity to the intervention using fidelity checklists (Supplementary material), administrative record review, and audio recordings of all home visits. All home visits were digitally audio recorded and transcribed in Spanish. One member of the study team [ER] coded administrative records from all home visits (27 total) and listened to a random subset of audio recordings of all home visits for 16 participants. Using a checklist, we evaluated domains related to delivery of the intervention components, as well as rapport building, active listening, and whether the nurses designated time to answer questions.

We defined dose as the number of contacts (in-person or phone) experienced by the participants. We calculated dose in terms of contacts with nurse managers and data collectors separately and combined.

### 2.5 Data analysis

#### 2.5.1 Statistical analysis

The study was powered to detect a 2-point difference in ACT score with 80% power and a 5% significance level, accounting for 9% attrition (10). Demographic and clinical characteristics of the study population were summarized and compared between the intervention and control arms using Student t-tests with unequal variances for continuous variables and Chi square or Fisher exact tests for categorical variables. ACT, ARMS-7, PAQLQ, PACQLQ and PHQ-9 scores (continuous) were summarized for each arm at baseline and 6 months and within group changes were compared using paired t-tests. All outcomes were compared at 6 months using unadjusted and multivariable linear and logistic regression models, as appropriate. Three adjusted models are presented and included: (1) adjustment for baseline score; (2) adjustment for baseline score, age at study entry, sex and disease severity; and (3) adjustment for age at study entry, sex and disease severity. We did not apply specialized techniques such as imputation to handle missing data; rather, analyses were conducted on available data. All analyses were conducted using STATA Version 18.0 (StataCorp, College Station, TX, United States).

#### 2.5.2 Analysis of qualitative data

In-depth interviews and audio recordings of nurse visits were digitally recorded and transcribed verbatim by a professional, external transcriber. Two team members (ERH, SLP) initially reviewed three transcripts to generate a preliminary set of codes through an inductive process. We used the constant comparative method to identify common themes across interviews that were most relevant to understanding caregiver perception of effectiveness, intervention engagement, acceptability, feasibility, and fidelity of the intervention from the perspectives of interviewees (19). A similar process was carried out with field notes from nurse managers. One team member (ERH) then applied the codes to all transcripts using Dedoose qualitative research software (20). All transcriptions were analyzed in the original language (Spanish) by native or fluent Spanish speakers.

### 2.6 Dissemination plans

We will disseminate our results within the local public health system, as Dr. Romani has a leadership role within the Ministry of Health epidemiological unit in the local area. It is accurate that rescue therapy is easily obtained, but controller therapy is not readily accessible or affordable. Thus, we will disseminate our findings to local and regional Ministry of Health authorities and professional networks, emphasizing that the lack of coverage and regular availability of inhaled corticosteroids in public insurance is a key bottleneck that needs to be addressed (e.g., by including controller therapy in benefits packages and ensuring reliable procurement and stocking). At the programmatic level, our nurse-delivered educational materials and visit guides can serve as a practical tool for primary care teams who already conduct home visits with their assigned families from their community.

### 2.7 Ethics approval statement

The trial protocol was approved by the Institutional Review Boards at the Johns Hopkins School of Medicine, Asociación Benéfica PRISMA, and Cayetano Heredia Hospital. Study personnel explained the goals of the study to both children and caregivers prior to enrollment. We obtained written informed consent and assent from caregivers and children, respectively. This behavioral study posed minimal risk to participants, and while we monitored for adverse events and had systematic reporting requirements in place, no adverse events occurred during this study.

## 3 Results

### 3.1 Participant characteristics

**Figure 2:**
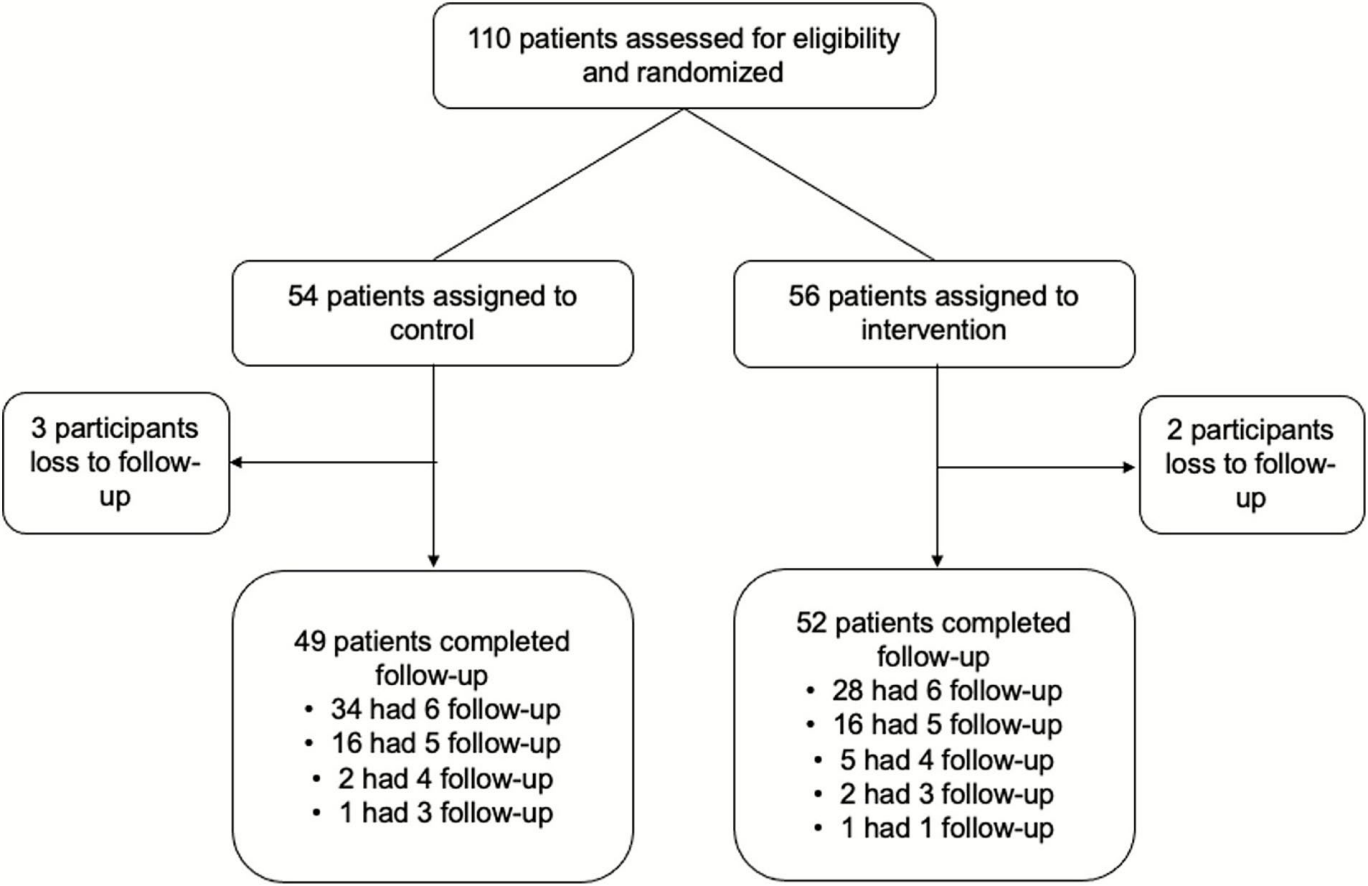
CONSORT diagram.

We enrolled and consented a total of 110 child-caregiver dyads. We randomized participants to two arms: 54 to intervention, 56 to control. Two intervention and three control participants were lost to follow-up (Figure 2); for intervention, one was no longer interested in participating, and the other moved to a different district; for control, all three could not be located. Overall, the mean age was 8.8 (SD, 2.27) years and 63 (62.4%) were male. All measured categories were similar in both groups, including ACT score, at baseline. Full characteristics of individuals who completed follow-up are shown in Table 2.

**Table 2:** Baseline characteristics of the intervention and control arms who completed follow-up.

Demographic characteristics	Overall (*n* = 101)	Intervention (*n* = 49)	Control (*n* = 52)	*p*-value
Age in years, mean ± SD	8.8 ± 2.27	8.8 ± 2.27	8.8 ± 2.46	0.886
Sex, n (%)				
Male	63 (62.4)	31 (63.3)	32 (61.5)	0.858
Female	38 (37.6)	18 (36.7)	20 (38.5)	
Insurance, n (%)				
Yes	91 (90.1)	44 (89.8)	47 (90.4)	0.921
No	10 (9.9)	5 (10.2)	5 (9.6)	
Parents education, n (%)				
Mother completed secondary school	79 (78.2)	37 (75.5)	42 (80.8)	0.522
Father completed secondary school	81 (80.2)	41 (83.7)	40 (76.9)	0.395
Monthly household income (Peruvian Nuevo Sol), n (%)				0.365
<=800 (204 USD)	34 (33.3)	17 (34.7)	17 (32.1)	
800–1,500 (205 to 384 USD)	50 (49.0)	21 (42.9)	29 (54.7)	
1,500 + (385 USD or more)	18 (17.7)	11 (22.5)	7 (13.2)	
Family and exposure history, n (%)				
Household member smokes	12 (11.9)	6 (12.2)	6 (11.5)	0.913
Parental history of asthma	27 (26.7)	11 (22.5)	16 (30.8)	0.345
Sibling history of asthma	17 (16.8)	8 (16.3)	9 (17.3)	0.895
Clinical characteristics				
Asthma severity, n (%)				0.502
Mild intermittent	2 (2.0)	0 (0.0)	2 (3.9)	
Mild persistent	18 (17.8)	9 (18.4)	9 (17.3)	
Moderate persistent	72 (71.3)	37 (75.5)	35 (67.3)	
Severe persistent	9 (8.9)	3 (6.1)	6 (11.5)	
ACT score, mean ± SD	19.1 ± 4.15	19.3 ± 3.80	18.9 ± 4.49	0.595
Healthcare Utilization, n (%)				
One or more emergency room visits in previous 12 months	91 (90.1)	42 (85.7)	49 (94.2)	0.152
One or more hospitalizations in previous 12 months	30 (29.7)	17 (34.7)	13 (25.0)	0.287
Followed medical treatment for asthma control (e.g., inhaled corticosteroids) at any point prior to the study	49 (48.5)	25 (51.0)	24 (46.2)	0.625

### 3.2 Effectiveness outcomes (clinical effectiveness, perceived effectiveness, engagement with the intervention)

#### 3.2.1 Differences in asthma control and healthcare utilization by arm

**Figure 3:**
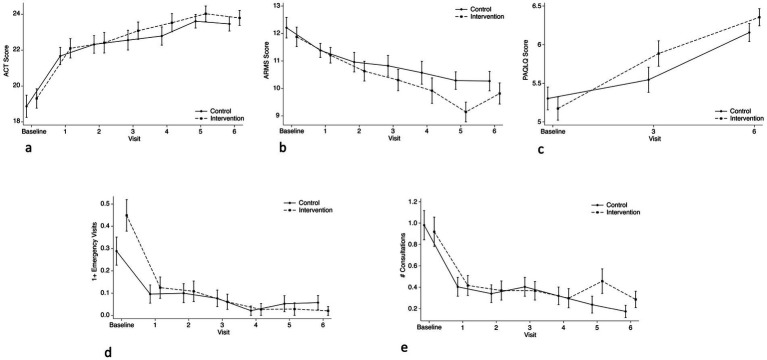
Comparison of changes in child outcomes between intervention and control arms across study visits. **(a)** ACT means from control and intervention group, both increased during follow-up. No difference among groups. **(b)** ARMS-7 mean scores, no statistical difference up to month 5 of follow-up; control 10.3 (1.96) and intervention 9.1 (2.13), *p* = 0.020. **(c)** PAQLQ score means both increased during follow-up. No difference among groups. **(d)** Emergency visit proportions from control and intervention group, both decreased during follow-up. No difference among groups. **(e)** Physician consultations proportions. A statistically significant difference is seen at follow-up month 5 (*p* = 0.048).

ACT scores did not differ significantly between intervention and control arms at any point during follow-up, including at 6 months (Figure 3a). ACT score increased by 4.49 (95% CI: 3.05–5.92) and 4.60 (95% CI: 3.22–5.99) points in the intervention and control arm, respectively, between baseline and 6 months (Table 3). ACT score also increased by 2.80 (95% CI: 1.67–3.93) and 2.61 (95% CI: 1.36–3.86) between baseline and the one-month visit in the intervention and control arm, respectively. Similarly, we did not observe a statistically significant difference in emergency department (ED) visits at any point in either arm (Figure 3d). Proportions of participants who had an ED visit and physician consultation decreased in both arms between baseline and 6 months (Figures 3d,e). No hospitalizations were reported during the follow-up period.

**Table 3:** Changes in child and caregiver outcomes between baseline and 6 months.

Outcome	Change, Intervention Arm (95% CI), (*n* = 54)			Change, Control Arm (95% CI), (*n* = 56)		
Child	Baseline mean (SD)	6 months mean (SD)	Mean difference (95% CI)	Baseline mean (SD)	6 months mean (SD)	Mean difference (95% CI)
ACT score (higher score = better outcome)	19.3 (3.80)	23.8 (2.89)	4.49 (3.05, 5.92)	18.9 (4.49)	23.5 (2.86)	4.60 (3.20, 5.99)
ARMS-7 (lower score = better adherence)	11.9 (2.47)	9.8 (2.71)	−2.06 (−3.10, −1.02)	12.2 (2.70)	10.3 (2.54)	−1.94 (−2.72, −1.16)
PAQLQ-Mini	5.2 (1.05)	6.4 (0.77)	1.18 (0.85, 1.52)	5.3 (1.07)	6.2 (0.84)	0.85 (0.55, 1.16)
Caregiver						
PACQLQ	4.8 (1.36)	6.3 (0.73)	1.49 (1.07, 1.91)	5.0 (1.23)	6.1 (1.01)	1.16 (0.84, 1.47)
PHQ-9	6.0 (5.10)	2.7 (3.28)	−3.37 (−4.91, −1.83)	5.6 (4.80)	2.4 (3.42)	−3.19 (−4.40, −1.99)

ACT, Asthma Control Test (lower score indicates poorer asthma control); ARMS7, Adherence to Refills and Medications Scale [lower scores represent better adherence to inhaled corticosteroid (ICS) treatment]; PAQLQ, Pediatric Asthma Quality of Life Questionnaire; PACQLQ, Pediatric Asthma Caregiver’s Quality of Life Questionnaire (higher score indicates better QOL); PHQ-9, Patient Health Questionnaire-9 (higher scores indicating a higher presence of depressive symptoms).

#### 3.2.2 Differences in medication adherence and disease-specific quality of life

We did not observe a statistically significant difference in ARMS-7 score or PAQLQ score between arms at 6 months (Figure 3b). ARMS-7 scores decreased (improved adherence) in each group and PAQLQ scores increased (improved QOL) significantly between baseline and 6 months (Figure 3c; Table 3).

#### 3.2.3 Caregiver quality of life and depressive symptoms

**Figure 4:**
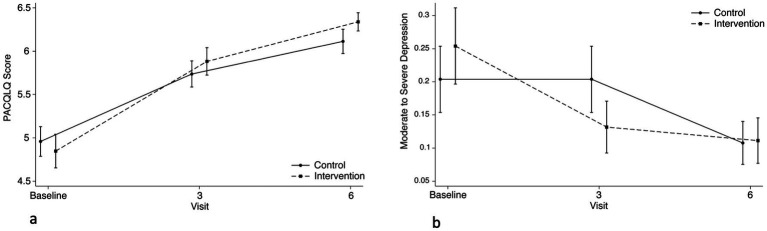
Comparison of changes in caregiver outcomes between intervention and control arms across study visits. **(a)** PACQLQ caregiver scores in caregivers. No difference between groups. **(b)** Proportion of caregivers with moderate-to-severe depression per PHQ > 10. No difference between groups.

We did not observe a statistically significant difference in caregiver quality of life (PACQLQ) or depressive symptoms (PHQ-9) between arms at 6 months (Figures 4a,b). We did observe a statistically significant increase (improved QOL) in PACQLQ score decrease in PHQ-9 score (reduced depressive symptoms) between baseline and 6 months (Table 3).

#### 3.2.4 Secondary regression analyses for differences between arms at 6 months

**Figure 5:**
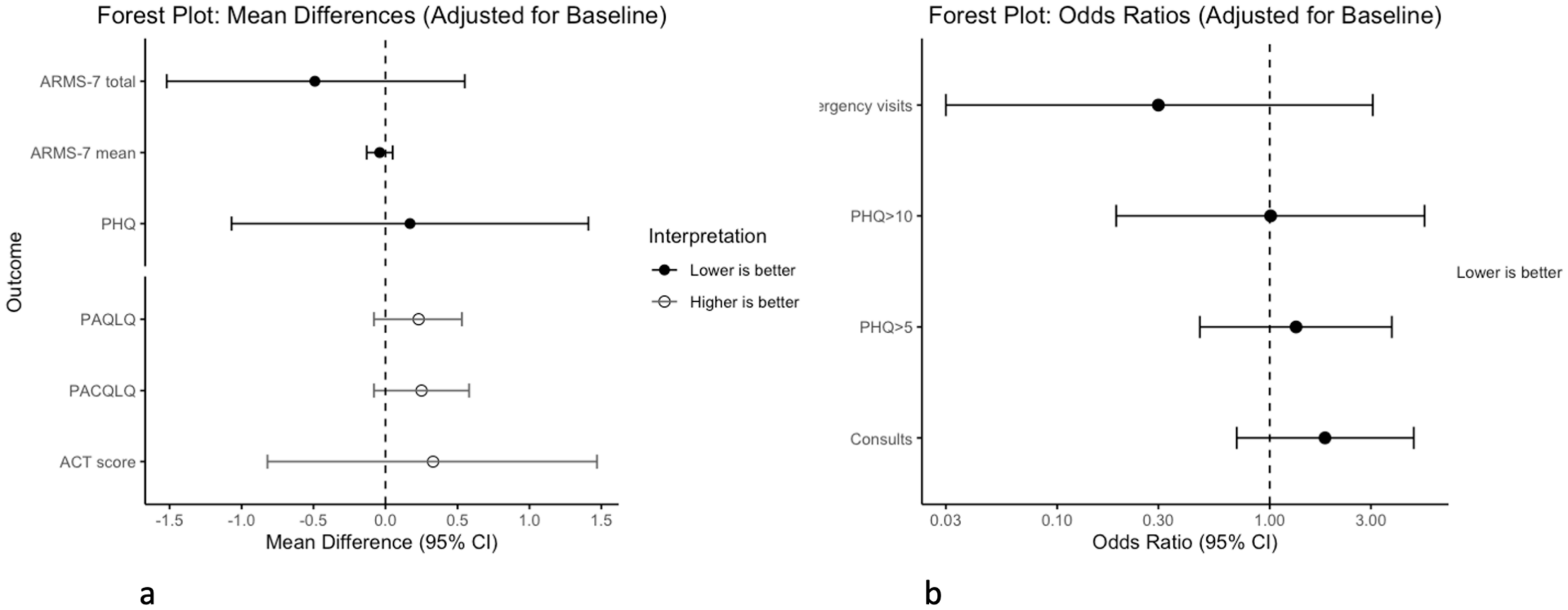
Results of regression analyses comparing intervention to control at 6 months. **(a)** Models for ACT (higher scores indicate better asthma control), ARMS-7 (lower scores indicate better adherence), PAQLQ (higher scores indicate better QoL), PACQLQ (higher scores indicate better QoL), and PHQ (lower scores indicate fewer depressive symptoms) are linear regression analyses. **(b)** Models for emergency visits, consults, PHQ > 5, and PHQ > 10 are logistic regression analyses.

In Figure 5, we display regression analyses showing differences for various outcomes between intervention and control arms at 6 months. We observed no differences in any outcomes between arms in either unadjusted or adjusted analyses. Details of the regression analyses can be found in Supplementary Table S2.

#### 3.2.5 Caregiver perception of effectiveness

Many caregivers in the intervention arm of the study expressed that they observed meaningful improvements in their children’s quality of life and asthma control. Some caregivers also expressed feeling less anxiety about their children’s asthma, suggesting that the sense of improvements in quality of life may have extended to many caregivers, as well.

*With respect to the visits, really good because it has informed us about how to use the [inhaler] and all that.…I’ve seen improvements, I no longer live constantly ready to go to the emergency room...there will always be some worry left, if I hear him coughing, but now…he gets over an attack quickly and he is learning how to control his own [asthma] and all that.* Caregiver of 6-year old boy, intervention.

Many caregivers in the control group also perceived improvements in asthma control, due largely to the consistent follow-up visits and calls from the data collection team. Caregiver(s) in the control group also expressed feeling less anxiety through their participation.

*I feel calmer. I can sleep…before joining this program, I couldn’t sleep. I woke up every time…to look at his chest, give him a pillow, feel him to make sure he was breathing, [now] he’s calmer, he can do more things that he couldn’t before.* Caregiver of 6-year old boy, control arm.

### 3.3 Engagement with the intervention

#### 3.3.1 Action plan

Caregivers expressed that the action plan was helpful for generally supporting their child in identifying symptoms and requesting assistance.

*The action plan is…so that he doesn’t get taken by surprise, meaning, he already has his inhaler, the phone numbers [he needs] in case something happens…in the [action] plan, red is because he’s sick, yellow is because he is intermediate, and the green…is that he is well.* Caregiver of 8-year old boy, intervention arm.

Still, many caregivers’ understandings of the nuance of each zone did not align with their intended definitions. For example, one caregiver describes her daughter using the concepts of the action plan to request her medication. “Feeling a little bad” would, according to clinical guidelines, belong in the yellow zone instead of the red zone.

*The action plan, she knows [that] green is when she’s doing well, and when she gets to red she tells me mami I feel a little bad, she says my inhaler please, she knows now.* Caregiver of 6-year old girl, intervention arm.

One caregiver we interviewed could not define the various zones when prompted.

#### 3.3.2 Demonstration of inhaler technique

Conversations with caregivers and nurses suggest that the demonstration and feedback on inhaler technique was helpful. Most caregivers and children were able to recreate the steps for proper inhaler technique successfully.

#### 3.3.3 Education—trigger reduction

Caregivers and children engaged with the various components of the intervention in different ways. More than one caregiver reported making physical changes to their home environment to reduce triggers, even building new areas of their home out of materials such as concrete that would produce less dust.

*I had to build my house, before it was just machihembrado [prefabricated wood]. Now, for her wellbeing more than anything, [I made improvements to the house]. Because…being surrounded by dust, all of that was affecting her.* Caregiver of 6-year old girl, intervention arm.

#### 3.3.4 Education—super Niño

Engagement with the Super Niño educational materials was particularly strong with younger children.

*I think [Super Niño] did work, especially for children from 5 years to about 8 or 9. When they’re bigger… there was less interest. For the little kids, the drawings, they’re learning to read, so they were very interested in it and paid a lot of attention…we would read it together, we looked at the drawings…they even put in order the steps for using the inhaler.* Nurse manager.

### 3.4 Implementation and maintenance

#### 3.4.1 Acceptability

Overall, caregivers whose children received the intervention found it acceptable. Many caregivers expressed a great deal of gratitude for the opportunity to receive guidance and support from the nurse managers. Here, one caregiver expressed how having both the nurse manager and the data collectors checking in on them made them feel supported.

*No, for me it was interesting because I had never had a doctor that was always calling, asking how my son was doing…In other hospitals they don’t do that…they don’t ask for your number, or they do ask and never call….But you all, in comparison, …you’ve been constantly calling, the young man and young woman…they’ve been calling, visiting, I feel that was really good.* Caregiver of 6-year old girl, intervention arm.

One woman in the control group also expressed that she places more trust in her child’s physician after participating in the program.

*Because now at least I know, I trust the doctor more, you all have helped me so much and I know it has been good for me.* Caregiver of 11-year old boy, control arm.

Caregivers were overwhelmingly grateful for the inhalers that they received free of charge. One caregiver perceived that the medications prescribed and provided to her child worked better than the medicines previously prescribed and obtained through the health system.

*Maybe it was the [formulation] that you have, I don’t know, because [my child] was on preventive medications with the budesonide, but…it didn’t work at all, but with the [combination inhaler], that has calmed [my child’s asthma] a lot.* Caregiver of 6-year old girl, control arm.

Several caregivers in the control arm of the study expressed their gratitude for the support and check-ins provided by the data collectors. One participant explained how the questions related to asthma control were helpful. It also gave the caregivers a sense of accountability.

*You all have helped me because, like I was saying, you’ve been calling to make me remember, knowing I had to give an update.* Caregiver of 12-year old girl, control arm.

Nurse managers also found the work gratifying and felt that their work was really making a difference in the lives of the families they were working with and the health of the children they visited.

*For me it was a really beautiful [experience], I’ve always worked in primary care, here in Peru we have…several functions ranging from the community to the hospital level, but I’ve always been most interested in the community part, you know? So, I found the experience really good, since I had [the opportunity] to understand the realities of each family, of every child, their habits, their day to day, their studies, their family. So for me as I’ve always said, I liked going to visit them, see how they were doing, and every time they achieved something, I felt really happy for them.* Nurse manager.

Nurse managers also found the training helpful and felt that their expertise as nurses served them greatly in their role providing health education, patient navigation, and social support. Nurses had prior training which made them very well suited for the position.

*I think that [the training] was good. The thing is that we [as nurses] have prior training, from being a nurse, going out into the community and all that…that really helped us do our job…at first we didn’t know [how to deliver the program], but with the training that we had, with all the guidelines…, and there were also things that happened, for example, with different families, and we learned…with experience.* Nurse manager.

#### 3.4.2 Feasibility

Nurse managers described willingness to participate from most families mainly related to the potential benefits of information, support and medication access to their children. Nonetheless, several challenges related to feasibility appeared. First, it took some time for trust and rapport to build between the participating families and the nurse managers, especially considering the close engagement required and the regular home visits. Families often had many time constraints, which presented challenges for scheduling visits and maintaining long-term engagement. One nurse manager also mentioned the logistical challenge of the home visits, including distance and lack of transportation. Although some households were located in neighborhoods that were distant or difficult to access, nurse managers and data collectors were generally able to reach them without major difficulties.

Many families also experienced social and structural challenges, such as poverty, low literacy, and competing priorities that affected their ability to fully engage in the program.

*I think that, like I was saying to you, I think that the [caregiver] was more concerned maybe in having enough money to subsist, that was the most important thing to her, because during that time she did not have stability…she was so stressed, so overwhelmed that…the most important thing to her was to find money to survive.* Nurse manager.

*I had one mother who it seemed didn’t know how to read…or parents that had only completed primary school. In those cases, to use the “Asma card” or any of the other instruments, we had to [adapt] based on the needs of each family.* Nurse manager.

The cost of medications is another well-documented and critical barrier to accessing evidence-based asthma care in low-resource communities in Lima, outside of the context of the research study.

*Yes well it’s a lot, one has to be brave, positive with [one’s children]. The [combination inhaler] that I bought, look when they prescribed it to me…one time I bought the [combination inhaler] and it cost 150 [soles]. The doctor also prescribed [one that] was 300 soles and it wasn’t even enough for a month…that’s 500 soles, imagine that, my God.* Caregiver of 10-year old boy, intervention arm.

Caregivers overwhelmingly expressed gratitude for the medications that were provided to all participants free of charge.

*“Well, since the program began…we have noticed a lot of improvement. [Before] she was good—bad—good--bad, you know?...I think that two things [helped my daughter]. The control visits in the hospital with the doctor and also, thank you, I don’t know if this was just a coincidence or luck or what not that we crossed paths, just when I went to her control visit and I could access this medicine, you know?* Caregiver of 12-year old girl, intervention arm.

### 3.5 Fidelity and dose

We present the results of the fidelity checklist in Supplementary Table S3. Overall, fidelity to the intervention protocol was high across all domains. In Supplementary Table S4, we present the mean number of visits (dose) from nurse managers and data collectors in each arm. Intervention arm participants experienced approximately twice the number of total contacts as control arm participants.

## 4 Discussion

We conducted a hybrid type 2 effectiveness-implementation individually randomized controlled trial to evaluate the effectiveness and implementation of a multi-faceted intervention package aimed at improving asthma control and quality of life in children with asthma. We used mixed methods to evaluate trial outcomes, guided by the RE-AIM framework. Both the intervention and control arms saw improvements in asthma control, disease-specific quality of life, and the frequency of ED visits at 6 months compared to baseline. The biggest improvements in both arms occurred between baseline and the first visit. For ACT score, the change between baseline and first visit approached the minimal clinically important difference (MCID) of 3 points (24) and the change between baseline and 6 months follow-up exceeded the MCID. However, we found no statistically significant differences in asthma control score or other effectiveness measures between arms at 6 months. Results from in-depth interviews suggest that most caregivers in both the intervention and control arms perceived an improvement in asthma control and overall wellbeing for their children. Many caregivers also reported feeling less anxious about their children’s asthma. Additionally, both caregivers and nurse managers found the intervention acceptable and professionally gratifying. Retention was high, with over 95% completing the study in both arms.

While we did not find differences in outcomes between study groups at 6 months, we did see improvements in both arms between baseline and 6 months. Families in both arms received controller medications free of charge, delivered directly to their homes. Providing both financial and physical access to these medications likely contributed greatly to these improvements. Notably, the greatest improvements occurred between baseline and 1 month of follow-up (Figures 3a–d). Still, we should exercise caution in minimizing the role of seasonality as a confounding factor, as the six-month duration of follow-up limits our ability to draw definitive conclusions about its impact.

Our results suggest that other factors related to the intervention may also have contributed to the improvements observed in both arms. While caregiver quality of life and mental health improved in both arms, caregivers from both arms also expressed feeling supported and less anxious as a direct result of the program. While caregivers in the control arm did not receive monthly visits from nurse managers, they nevertheless felt supported by the regular calls and visits from data collectors, who asked questions about their children’s asthma control, medication use, and quality of life, and their mental health and quality of life. These data collectors developed a level of rapport with the families, who in turn felt supported and cared for. These results support the idea that data collection activities may have functioned as a form of intervention that may have contributed to the improvements observed in both arms.

A key theme that emerged from our data was the critical role of poverty and medication accessibility (cost and availability) on a family’s ability to support their children’s asthma management. In Peru, asthma medications are available for free to patients enrolled in the national insurance program (Seguro Integral de Salud). However, access to these medications largely depends on their availability at individual public/private pharmacies and community health posts. Short-term medications like salbutamol/albuterol are often available in both public and private pharmacies (21). However, preventive medications like ICS or long-acting beta agonists, as well as combination inhalers, are scarce (3). Even if these medications are available, they are often out of reach financially for families living in poverty. Pricing disparities also exist, with the cost of these medications increasing significantly in rural areas (21). Thus, in this context, simply providing medications to participants may by itself have been motivation enough to initiate and continue treatment. From our cohort, 61 (51.5%) individuals had never been on controller medications before joining the study despite having persistent asthma. These results highlight the critical need to ensure the availability and affordability of controller medications in LMIC contexts such as Peru. Multi-month dosing programs, which are recommended for delivering antiretroviral therapy in low-resource contexts (22), could reduce logistical hurdles to maintaining a continuous supply of controller medications for families.

**Figure 6:**
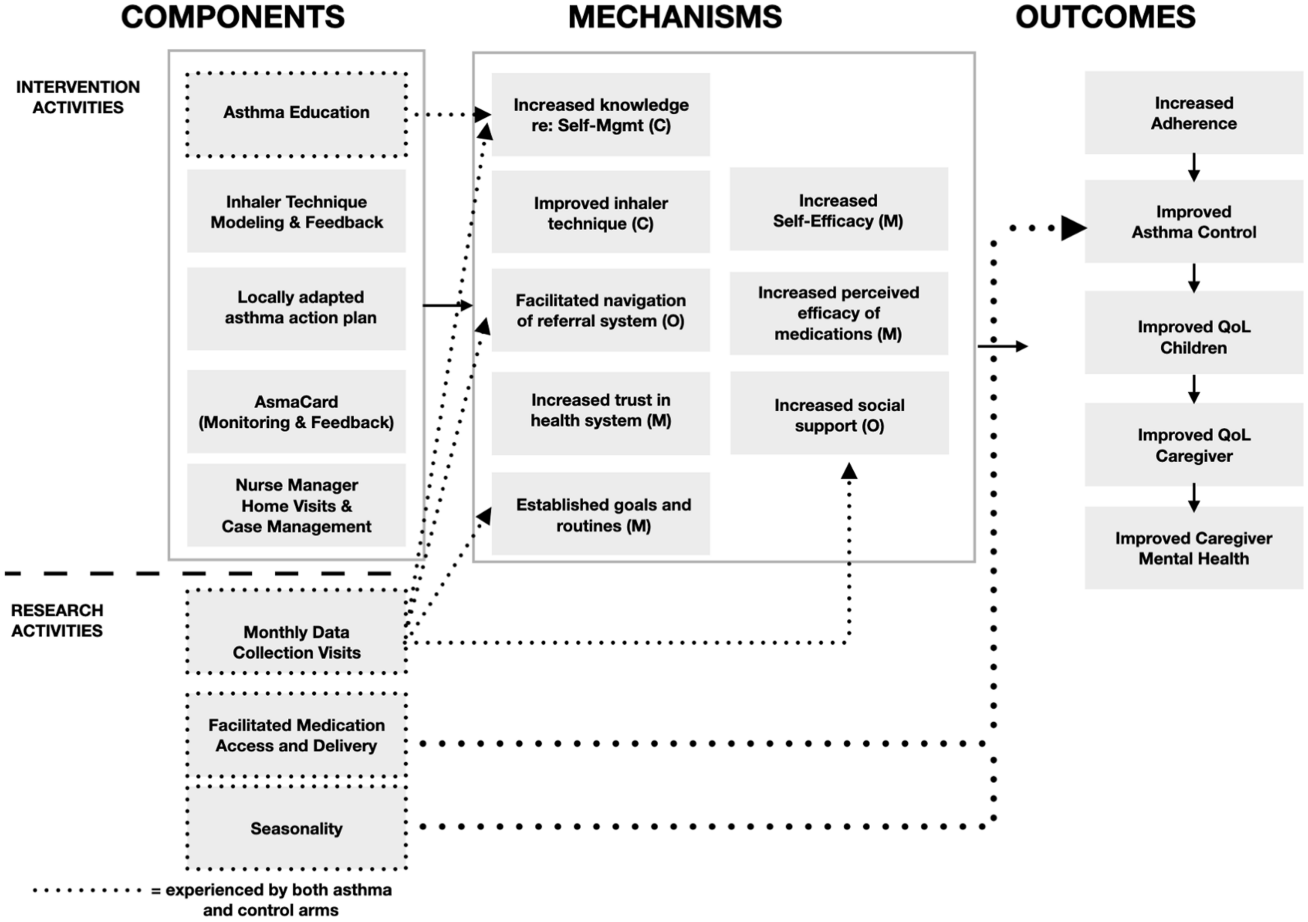
Updated theory of change.

In light of our findings, in Figure 6, we display a revised Theory of Change (ToC) for each arm that reflect the realities of data collection on the ground. Monthly data collection visits alone appeared to have targeted several key barriers, specifically, increased social support, improved self-efficacy, increased trust in the health system, and established goals and routines. Furthermore, provision and home delivery of asthma medications may have both improved self-efficacy and led to direct clinical improvements in adherence and improved asthma control and quality of life. Through these mechanisms, data collection activities may have contributed to improved outcomes in both arms. Seasonality is another critical factor that contributed to these improvements and is also reflected in the revised ToC. This ToC could be used to develop and evaluate a lighter-touch intervention, such as regular symptom monitoring, social support, and facilitated access to essential medications, may lead to improvements in asthma control, caregiver mental health, and social support in this context.

Nurse managers described several feasibility challenges, such as time constraints and logistical challenges, most of which were overcome within the context of the research project but could pose difficulties for integrating, scaling up, and sustaining the intervention within the existing healthcare infrastructure. We chose to employ nurses as managers and patient navigators for this study based on the expertise of leadership on our study team and feedback from nurses currently employed by the health system. We felt strongly that nurses had the best combination of healthcare delivery expertise, health education skills, patient navigation experience, and established community ties to be well-suited for this role. However, while the nurses in this study were employed full-time as research staff, nurses currently working within the health system would face competing demands and would likely be overburdened with other responsibilities. To improve feasibility, potential strategies include recruiting community health workers for specific duties under the supervision of a nurse manager, co-locating patient navigation services within clinic settings, or incorporating virtual or hybrid options for providing nurse manager support.

This study has several strengths. First, this is one of few studies to design and implement an asthma self-management intervention trial in a LMIC, which face unique or exacerbated barriers to asthma control. Second, the intervention was designed using a strong theoretical framework and was grounded in strong formative research and developed using iterative pilot testing (10). Our dual focus on both effectiveness and implementation allowed us not only to detect difference in clinical outcomes and quality of life, but also to document and describe the mechanisms and processes underlying these outcomes.

This study also has important limitations. First, the trial duration was limited to 6 months due to funding limitations, which restricted our ability to assess long-term outcomes and account for secular trends among patients and caregivers. Second, while we chose to provide medications to both study arms due to ethical considerations and local preferences, and following extensive discussion within our team and with local stakeholders, it may have ultimately limited our ability to evaluate the effectiveness of the intervention between arms at 6 months. While our approach did directly address structural barriers such as accessibility of healthcare providers and facilities and the complexity of the referral process, it was not focused on integrating and implementing an intervention within an existing health system. To realize improvements in childhood asthma outcomes at scale, a multi-level of systems-oriented approach to implementation would be needed.

Our experience underscores the importance of measuring implementation outcomes, even in studies that may be focused primarily on efficacy or effectiveness. Capturing rich implementation data enabled us to parse the mechanisms that may have led to improvements in both study arms despite not observing differences between arms. Furthermore, our experience highlights the value of making explicit what is usually considered implicit – recognizing how the research context interacts with and influences intervention delivery, the recipients of an intervention, and study outcomes. Similar to the anthropological concept of positionality (23), the pursuit of objectivity can sometimes hinder us from reflecting on our position as researchers. Despite our efforts to the contrary, research cannot be carried out in vacuum. Intervention research, even when focused on efficacy or effectiveness, should carefully assess how research activities, power dynamics, and the observer effect may influence outcomes. Finally, though outside the scope of this article, a cost analysis of the intervention its recommended for future studies, especially in limited-resource settings such as ours.

## 5 Conclusion

Our study was one of few studies to implement and test an asthma supported self-management program for children in a LMIC, who experience unique challenges to achieving asthma control. Our study highlights the importance and potential impact of enhancing medication accessibility and affordability, including the need for structural solutions for improving asthma management in these contexts. Lastly, it underscores that all intervention studies, even those primarily focused on efficacy or effectiveness, can benefit from carefully documenting, measuring, and reporting implementation outcomes and processes.

## Data Availability

The raw data supporting the conclusions of this article will be made available by the authors, without undue reservation.

## Footnotes

1
https://doi.org/10.1186/s13063-020-4207-5
