# Supplementary material for: Effectiveness and implementation of a multi-faceted intervention to facilitate adoption of asthma self-management practices in Peruvian children and adolescents: a hybrid type 2 individually randomized controlled trial
Source: Front Public Health. 2026 Jan 13;13:1710746. doi: 10.3389/fpubh.2025.1710746 (PMC12835256; doi:10.3389/fpubh.2025.1710746)
Supplement: Supplementary file 1 [file Supplementary_file_1.pdf]

Supplementary Material

1 Supplementary Data

2 Supplementary Figures and Tables

Table S1. Results of regression analyses comparing intervention to control at six months. Models for ACT, ARMS-7, PAQLQ, and PHQ are linear regression analyses. Models for emergency visits, consults, PHQ>5, and PHQ>10 are logistic regression analyses.

	**Unadjusted**	**Adjusted for baseline**	**Adjusted for baseline and age, sex, severity**	**Adjusted for age, sex, severity**
Child				
ACT score	0.33 (-0.80, 1.47)	0.33 (-0.82, 1.47)	0.33 (-0.83, 1.49)	0.34 (-0.82, 1.49)
Emergency visits	0.34 (0.03, 3.39)	0.3 (0.03, 3.06)	0.33 (0.03, 3.46)	0.34 (0.03, 3.52)
Consults	1.91 (0.74, 4.94)	1.82 (0.70, 4.77)	1.82 (0.69, 4.78)	1.91 (0.74, 4.95)
ARMS-7 total	-0.45 (-1.49, 0.58)	-0.49 (-1.52, 0.55)	-0.49 (-1.53, 0.55)	-0.45 (-1.49, 0.59)
ARMS-7 mean	-0.04 (-0.12, 0.05)	-0.04 (-0.13, 0.05)	-0.04 (-0.13, 0.05)	-0.04 (-0.12, 0.05)
PAQLQ	0.2 (-0.12, 0.52)	0.23 (-0.08, 0.53)	0.23 (-0.08, 0.54)	0.2 (-0.11, 0.52)
Caregiver				
PACQLQ	0.23 (-0.13, 0.58)	0.25 (-0.08, 0.58)	0.25 (-0.09, 0.59)	0.22 (-0.13, 0.58)
PHQ	0.29 (-1.04, 1.61)	0.17 (-1.07, 1.41)	0.15 (-1.10, 1.39)	0.27 (-1.07, 1.61)
PHQ>5	1.38 (0.52, 3.70)	1.33 (0.47, 3.76)	1.32 (0.46, 3.78)	1.38 (0.51, 3.68)

PHQ>10	1.07 (0.20, 5.55)	1.01 (0.19, 5.36)	1.2 (0.20, 6.99)	1.11 (0.20, 6.18)

(ACT) Asthma Control Test; (ARMS7) Adherence to Refills and Medications Scale; (PAQLQ) Pediatric Asthma Quality of Life Questionnaire; (PACQLQ) Pediatric Asthma Caregiver's Quality of Life Questionnaire; (PHQ-9) Patient Health Questionnaire-9

Table S2. Results of fidelity checklist assessments of study visit records and audio recordings of study visits.

ACTIVITY	V0		V1		V2		V3		V4		V5		V6	
Baseline	Records	Audio	Records	Audio	Records	Audio	Records	Audio	Records	Audio	Records	Audio	Records	Audio
Health education – general concepts, signs and symptoms of asthma (flipchart, videos)	52/52 100%	15/15 100%	*	*	*	*	*	*	*	*	*	*	*	*
Delivery of materials and explanation (Super Niño, Asthma Card, Action Plan)	52/52 100%	15/15 100%	*	*	*	*	*	*	*	*	*	*	*	*
Action Plan														
Presentation and delivery of instrument	52/52 100%	15/15 100%	*	*	*	*	*	*	*	*	*	*	*	*
Explanation of asthma control zones	52/52 100%	15/15 100%	*	*	*	*	*	*	*	*	*	*	*	*
Learn alongside the child and caregiver how to determine what zone they are in	52/52 100%	15/15 100%	*	*	*	*	*	*	*	*	*	*	*	*
Explain which actions to take based on symptoms	52/52 100%	15/15 100%	*	*	*	*	*	*	*	*	*	*	*	*
Explain how to use the daily medication diary in the AsmaCard	52/52 100%	15/15 100%	*	*	*	*	*	*	*	*	*	*	*	*
AsmaCard Tool														

Presentation and delivery of instrument	52/52 100%	15/15 100%	*	*	*	*	*	*	*	*	*	*	*	*
Respond to ACT questionnaire and calculate score	52/52 100%	10/15 67%												
Record details of prescription from physician	52/52 100%	10/15 67%	*	*	*	*	*	*	*	*	*	*	*	*
Record personal and medical information	52/52 100%	10/15 67%	*	*	*	*	*	*	*	*	*	*	*	*
Record known asthma triggers	52/52 100%	13/15 87%	*	*	*	*	*	*	*	*	*	*	*	*
Ask families to save instrument and keep in safe place	52/52 100%	15/15 100%	*	*	*	*	*	*	*	*	*	*	*	*
Delivery and demonstration of use of inhaler and spacer	52/52 100%	15/15 100%	*	*	*	*	*	*	*	*	*	*	*	*
Follow-up	V0		V1		V2		V3		V4		V5		V6	
Check-in on use of instruments	*	*	48/51 94.1%	12/14 87.5%	47/51 92.1%	13/14 98%	36/49 73.4%	8/12 66.6%	35/45 78%	3/3 100%	20/34 59%	3/3 100%	7/22 31.9%	3/3 100%
Evaluation of steps in use of inhaler/spacer	*	*	48/51 94.1%	12/14 87.5%	47/51 92.1%	13/14 98%	36/49 73.4%	11/12 97.6%	35/45 78%	3/3 100%	20/34 59%	3/3 100%	7/22 31.9%	3/3 100%
Reinforce asthma concepts related to their progress, self-management, triggers	*	*	48/51 94.1%	13/14 98%	*	*	*	*	*	*	*	*	*	*
Education about the types of inhalers (short- vs. Long-acting)	*	*	48/51 94.1%	13/14 98%	*	*	*	*	*	*	*	*	*	*

Provide feedback on adherence and self-management, adapted to the needs of the family and child	*	*	*	*	47/51<br>92.1%	13/14<br>98%	45/49<br>92%	12/12<br>100%	40/45<br>89%	3/3<br>100%	20/34<br>59%	3/3<br>100%	7/22<br>31.8%	3/3<br>100%
Collect information about asthma management in school	*	*	*	*	*	*	15/49<br>31%	3/12<br>25%	*	*	*	*	*	*
Emphasize the empowerment of the caregiver and child	*	*	*	*	*	*	30/49<br>61.2%	9/12<br>75%	35/45<br>78%	3/3<br>100%	*	*	*	*
Health education depending on the family's specific questions or concerns	*	*	*	*	*	*	*	*	40/45<br>89%	3/3<br>100%	*	*	*	*
Education about potential complications and how to prevent them	*	*	*	*	*	*	*	*	*	*	20/34<br>59%	3/3<br>100%	*	*
Support on control of asthma triggers	*	*	*	*	*	*	*	*	*	*	15/34<br>44.1%	3/3<br>100%	*	*
Evaluation and feedback on concepts learned	*	*	*	*	*	*	*	*	*	*	*	*	7/22<br>31.8%	3/3<br>100%
Nurse manager skills	V0		V1		V2		V3		V4		V5		V6	
Good rapport	*	15/15<br>100%	*	15/15<br>100%	*	15/15<br>100%	*	15/15<br>100%	*	15/15<br>100%	*	12/12<br>100%	*	9/9<br>100%
Active listening	*	15/15<br>100%	*	15/15<br>100%	*	15/15<br>100%	*	15/15<br>100%	*	15/15<br>100%	*	12/12<br>100%	*	9/9<br>100%
Resolves questions	*	15/15<br>100%	*	15/15<br>100%	*	15/15<br>100%	*	15/15<br>100%	*	15/15<br>100%	*	12/12<br>100%	*	9/9<br>100%
30-45 minute visit	*	15/15<br>100%	*	15/15<br>100%	*	15/15<br>100%	*	15/15<br>100%	*	15/15<br>100%	*	12/12<br>100%	*	9/9<br>100%

Table S3. Dose of visit contacts experienced by intervention and control arms with nurse managers, data collectors, and both.

	**Total contacts with nurse manager**	**Total contacts with data collector**	**Total contacts**
**Intervention**	6.02	9.00	15.02
**Control**	1.00	6.23	7.23

2.1 Supplementary Figures

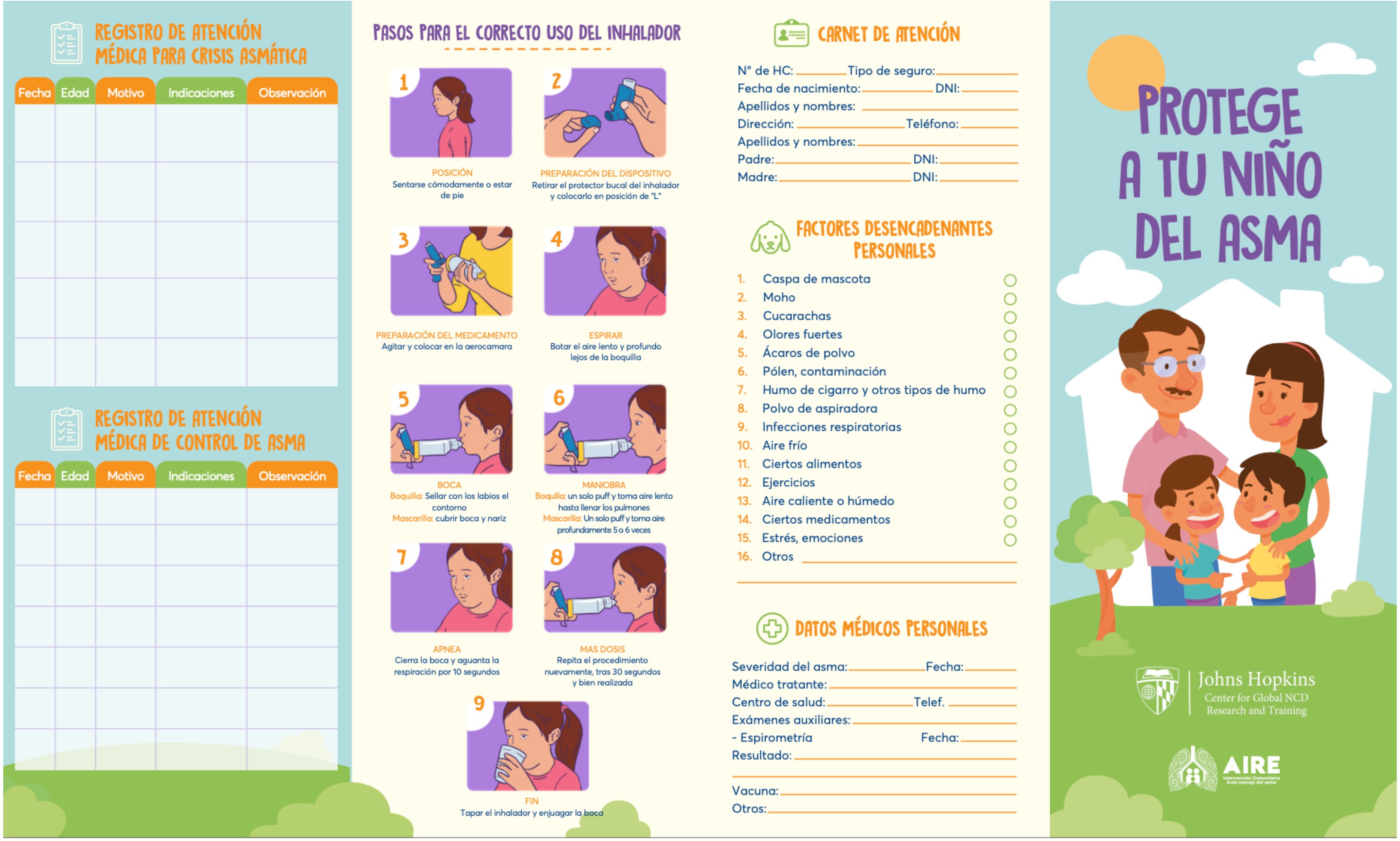

REGISTRO DE ATENCIÓN MÉDICA PARA CRISIS ASMÁTICA

Fecha	Edad	Motivo	Indicaciones	Observación

REGISTRO DE ATENCIÓN MÉDICA DE CONTROL DE ASMA

Fecha	Edad	Motivo	Indicaciones	Observación

PASOS PARA EL CORRECTO USO DEL INHALADOR

1. POSICIÓN
Sentarse cómodamente o estar de pie

2. PREPARACIÓN DEL DISPOSITIVO
Retirar el protector bucal del inhalador y colocarlo en posición de "L"

3. PREPARACIÓN DEL MEDICAMENTO
Agitar y colocar en la aerocamara

4. ESPIRAR
Botar el aire lento y profundo lejos de la boquilla

5. BOCA
Boquilla: Sellar con los labios el contorno
Mascarilla: cubrir boca y nariz

6. MANIOBRA
Boquilla: un solo puff y toma aire lento hasta llenar los pulmones
Mascarilla: Un solo puff y toma aire profundamente 5 o 6 veces

7. APNEA
Cierra la boca y aguanta la respiración por 10 segundos

8. MAS DOSIS
Repita el procedimiento nuevamente, tras 30 segundos y bien realizada

9. FIN
Tapar el inhalador y enjuagar la boca

CARNET DE ATENCIÓN

N° de HC: ________ Tipo de seguro: ________
Fecha de nacimiento: ________ DNI: ________
Apellidos y nombres: ________
Dirección: ________ Teléfono: ________
Apellidos y nombres: ________
Padre: ________ DNI: ________
Madre: ________ DNI: ________

FACTORES DESENCADENANTES PERSONALES

1. Caspa de mascota ○
2. Moho ○
3. Cucarachas ○
4. Olores fuertes ○
5. Ácaros de polvo ○
6. Pólen, contaminación ○
7. Humo de cigarro y otros tipos de humo ○
8. Polvo de aspiradora ○
9. Infecciones respiratorias ○
10. Aire frío ○
11. Ciertos alimentos ○
12. Ejercicios ○
13. Aire caliente o húmedo ○
14. Ciertos medicamentos ○
15. Estrés, emociones ○
16. Otros ________

DATOS MÉDICOS PERSONALES

Severidad del asma: ________ Fecha: ________
Médico tratante: ________
Centro de salud: ________ Telef. ________
Exámenes auxiliares: ________
- Espirometría Fecha: ________
Resultado: ________

Vacuna: ________
Otros: ________

PROTEGE A TU NIÑO DEL ASMA

Johns Hopkins
Center for Global NCD Research and Training

AIRE

GRÁFICA DE TENDENCIA DE CONTROL DEL ASMA

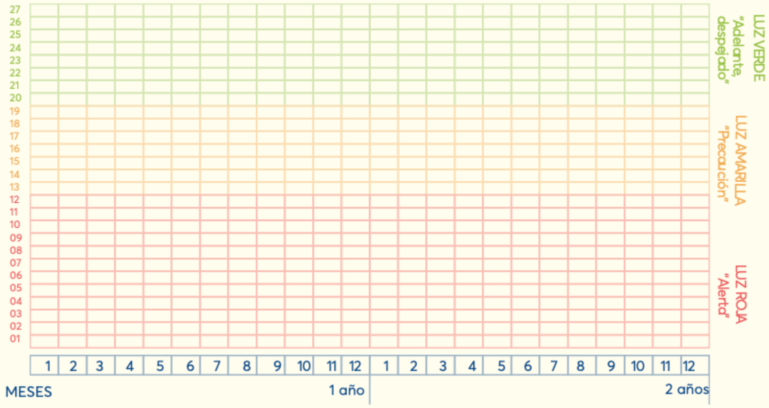

1 Los medicamentos de control a largo plazo o mantenimiento

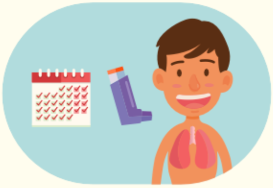

2 Ojo con los factores que desencadenan el asma

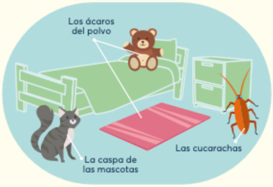

3 Los medicamentos de alivio rápido

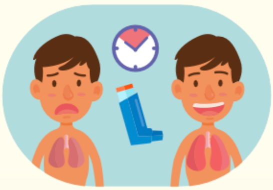

4 ¿Cúales son los síntomas comunes del asma?

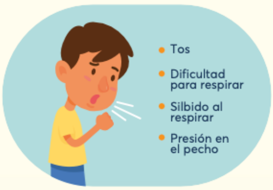

PRUEBA DEL CONTROL DEL ASMA INFANTIL PARA NIÑOS DE 5 A 11 AÑOS

CÓMO HACER LA PRUEBA DEL CONTROL DEL ASMA INFANTIL

PASO 1: Deje que su hijo responda las primeras cuatro preguntas (1 a 4). Si su hijo necesita ayuda para leer o comprender la pregunta, usted puede ayudar , pero deje que su hijo elija la respuesta. Responda las tres preguntas restantes (5 a 7) usted solo sin dejar que la respuesta de su hijo influya en sus respuestas.
No hay respuestas correctas o incorrectas.

PASO 2: Escriba el número de cada respuesta en la casilla de puntaje provista.

PASO 3: Sume las casillas de puntaje para obtener el total.

PASO 4: Lleve la prueba al médico para hablar sobre el puntaje total de su hijo.

19 O MENOS: Si su puntaje de su hijo es de 19 o menos, puede ser un indicio de que el asma de su hijo no está tan bien controlada como podría estarlo. Sin importar cúal sea el puntaje, lleve la prueba al médico para hablar sobre los resultados de su hijo.

PÍDELE A SU HIJO QUE RESPONDA ESTAS PREGUNTAS

PUNTAJE

1. ¿Cómo está tu asma hoy?

0	1	2	3
Muy mal	Mal	Bien	Muy bien

2. ¿Qué tipo de problema te causa el asma cuando corres , haces ejercicios o practicas deportes?

0	1	2	3
Un gran problema, no puedo hacer lo que quiero hacer	Es un problema y no me gusta	Un problema pequeño, pero está bien	No es un problema

3. ¿Toses por el asma?

0	1	2	3
Sí, todo el tiempo	Sí, la mayor parte del tiempo	Sí, un poco	No, nada

4. ¿Te despiertas durante la noche por el asma?

0	1	2	3
Sí, todo el tiempo	Sí, la mayor parte del tiempo	Sí, un poco	No, nunca

Responda las siguientes preguntas usted solo

5. Durante las últimas 4 semanas ¿Cuántos días su hijo tuvo síntomas de asma durante el día?

5	4	3	2	1	0
Ninguno	1 a 3 días	4 a 10 días	11 a 18 días	19 a 24 días	Todos los días

6. Durante las últimas 4 semanas ¿Cuántos días su hijo tuvo sibilancias durante el día debido al asma?

5	4	3	2	1	0
Ninguno	1 a 3 días	4 a 10 días	11 a 18 días	19 a 24 días	Todos los días

7. Durante las últimas 4 semanas ¿Cuántos días su hijo se despertó durante la noche debido al asma?

5	4	3	2	1	0
Ninguno	1 a 3 días	4 a 10 días	11 a 18 días	19 a 24 días	Todos los días

TOTAL

REGISTRO DE ATENCIÓN MÉDICA PARA CRISIS ASMÁTICA

Fecha	Edad	Motivo	Indicaciones	Observación

REGISTRO DE ATENCIÓN MÉDICA DE CONTROL DE ASMA

Fecha	Edad	Motivo	Indicaciones	Observación

PASOS PARA EL CORRECTO USO DEL INHALADOR

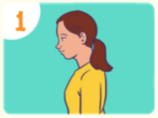

POSICIÓN
Sentarse cómodamente o estar de pie

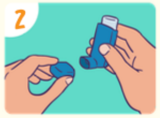

PREPARACIÓN DEL DISPOSITIVO
Retirar el protector bucal del inhalador y colocarlo en posición de "L"

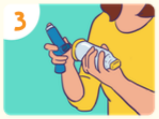

PREPARACIÓN DEL MEDICAMENTO
Agitar y colocar en la aerocamara

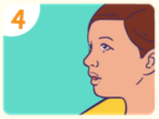

ESPIRAR
Botar el aire lento y profundo lejos de la boquilla

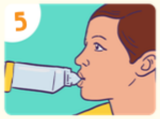

BOCA
Boquilla: Sellar con los labios el contorno
Mascarilla: cubrir boca y nariz

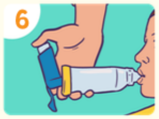

MANIOBRA
Boquilla: un solo puff y toma aire lento hasta llenar los pulmones
Mascarilla: Un solo puff y toma aire profundamente 5 o 6 veces

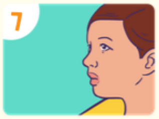

APNEA
Cierra la boca y aguanta la respiración por 10 segundos

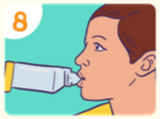

MAS DOSIS
Repita el procedimiento nuevamente, tras 30 segundos y bien realizada

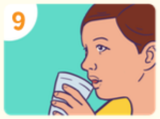

FIN
Tapar el inhalador y enjuagar la boca

CARNET DE ATENCIÓN

N° de HC: ________ Tipo de seguro: ________
Fecha de nacimiento: ________ DNI: ________
Apellidos y nombres: ________
Dirección: ________ Teléfono: ________
Apellidos y nombres: ________
Padre: ________ DNI: ________
Madre: ________ DNI: ________

FACTORES DESENCADENANTES PERSONALES

1. Caspa de mascota ○
2. Moho ○
3. Cucarachas ○
4. Olores fuertes ○
5. Ácaros de polvo ○
6. Pólen, contaminación ○
7. Humo de cigarro y otros tipos de humo ○
8. Polvo de aspiradora ○
9. Infecciones respiratorias ○
10. Aire frío ○
11. Ciertos alimentos ○
12. Ejercicios ○
13. Aire caliente o húmedo ○
14. Ciertos medicamentos ○
15. Estrés, emociones ○
16. Otros ________

DATOS MÉDICOS PERSONALES

Severidad del asma: ________ Fecha: ________
Médico tratante: ________
Centro de salud: ________ Telef. ________
Exámenes auxiliares: ________
- Espirometría ________ Fecha: ________
Resultado: ________

Vacuna: ________
Otros: ________

CONTROLEMOS EL ASMA

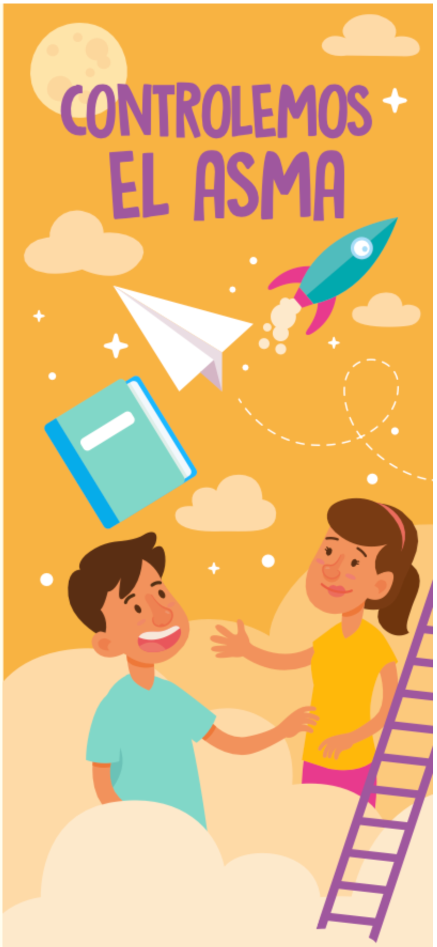

Johns Hopkins Center for Global NCD Research and Training
AIRE Intervención Comunitaria Auto-manejo del asma

GRÁFICA DE TENDENCIA DE CONTROL DEL ASMA

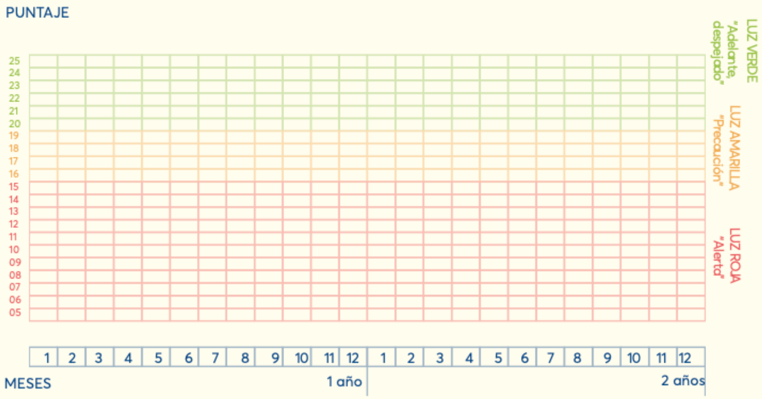

1 Los medicamentos de control a largo plazo o mantenimiento

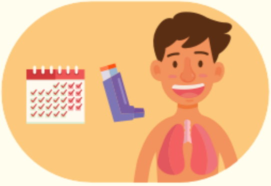

2 Ojo con los factores que desencadenan el asma

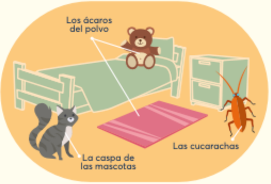

3 Los medicamentos de alivio rápido

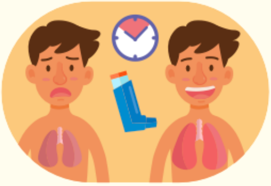

4 ¿Cúales son los síntomas comunes del asma?

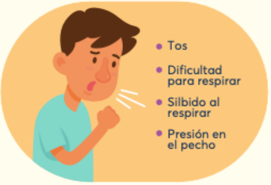

PRUEBA DEL CONTROL DEL ASMA PARA ADOLESCENTES DE 12 EN ADELANTE

PARA LOS PACIENTES

Tome la Prueba de Control del Asma (Asthma Control Test™ - ACT) para personas de 12 años de edad en adelante.

PASO 1: Anote el número correspondiente a cada respuesta en el cuadro de la derecha.
PASO 2: Sume todos los puntos en los cuadros para obtener el total.
PASO 3: Llévele la prueba a su doctor para hablar sobre su puntaje total.

PUNTAJE

1. En las últimas 4 semanas, ¿Cúanto tiempo le ha impedido su asma hacer todo lo que quería en el trabajo, en la escuela o en la casa?

1	2	3	4	5
Siempre	La mayoriá del tiempo	Algo del tiempo	Un poco del tiempo	Nunca

2. Durante las últimas 4 semanas ¿con qué frecuencia le ha faltado aire?

1	2	3	4	5
Más de una vez al día	Una vez por día	De 3 a 6 veces por semana	Una o dos veces por semana	Nunca

3. Durante las últimas 4 semanas, ¿con qué frecuencia sus síntomas del asma(respiración sibilante o un silbido en el pecho, tos, falta de aire, opresión en el pecho o dolor) lo/la despertaron durante la noche o más temprano de lo usual en la mañan

1	2	3	4	5
4 o más noches por semana	2 o 3 veces por semana	Una vez por semana	Una o dos veces	Nunca

4. Durante las últimas 4 semanas ¿Con qué frecuencia ha usado su inhalador de resc ate o medicamento en nebulizador (como albuterol)?

1	2	3	4	5
3 o más veces al día	1 o 2 veces al día	2 o 3 veces por semana	Una vez por semana o menos	Nunca

5. ¿Cómo evaluará el control de su asma durante las últimas 4 semanas?

1	2	3	4	5
No controlada, en absoluto	Mal controlada	Algo controlada	Bien controlada	Nunca

TOTAL

19 O MENOS: Si obtuvo 19 puntos o menos, es posible que su asma no esté tan bien controlada como podría. Hable con su médico.

PARA LOS MÉDICOS:

LA PRUEBA ACT:
- Ha sido convalidada clínicamente por espirmetría y evaluaciones de especialistas.
- Tiene el apoyo de la American Lung Association (Asociación Americana del Pulmón).
- Consiste en un breve cuestionario de 5 preguntas al que el paciente responde independientemente y que puede ayudarle al médico a evaluar el asma de sus pacientes durante las últimas 4 semanas.

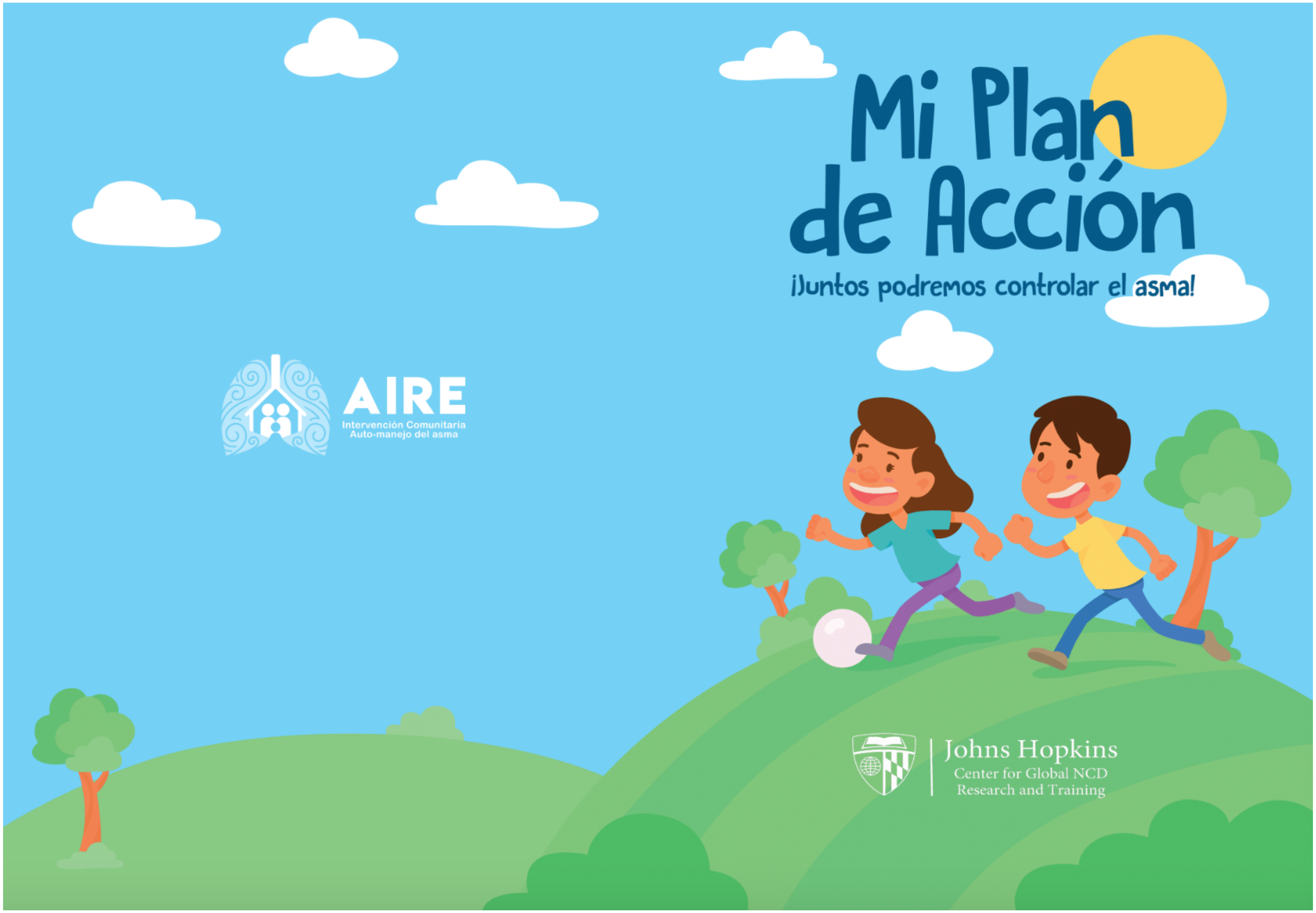
Mi Plan de Acción
¡Juntos podremos controlar el asma!
AIRE
Intervención Comunitaria
Auto-manejo del asma
Johns Hopkins
Center for Global NCD
Research and Training

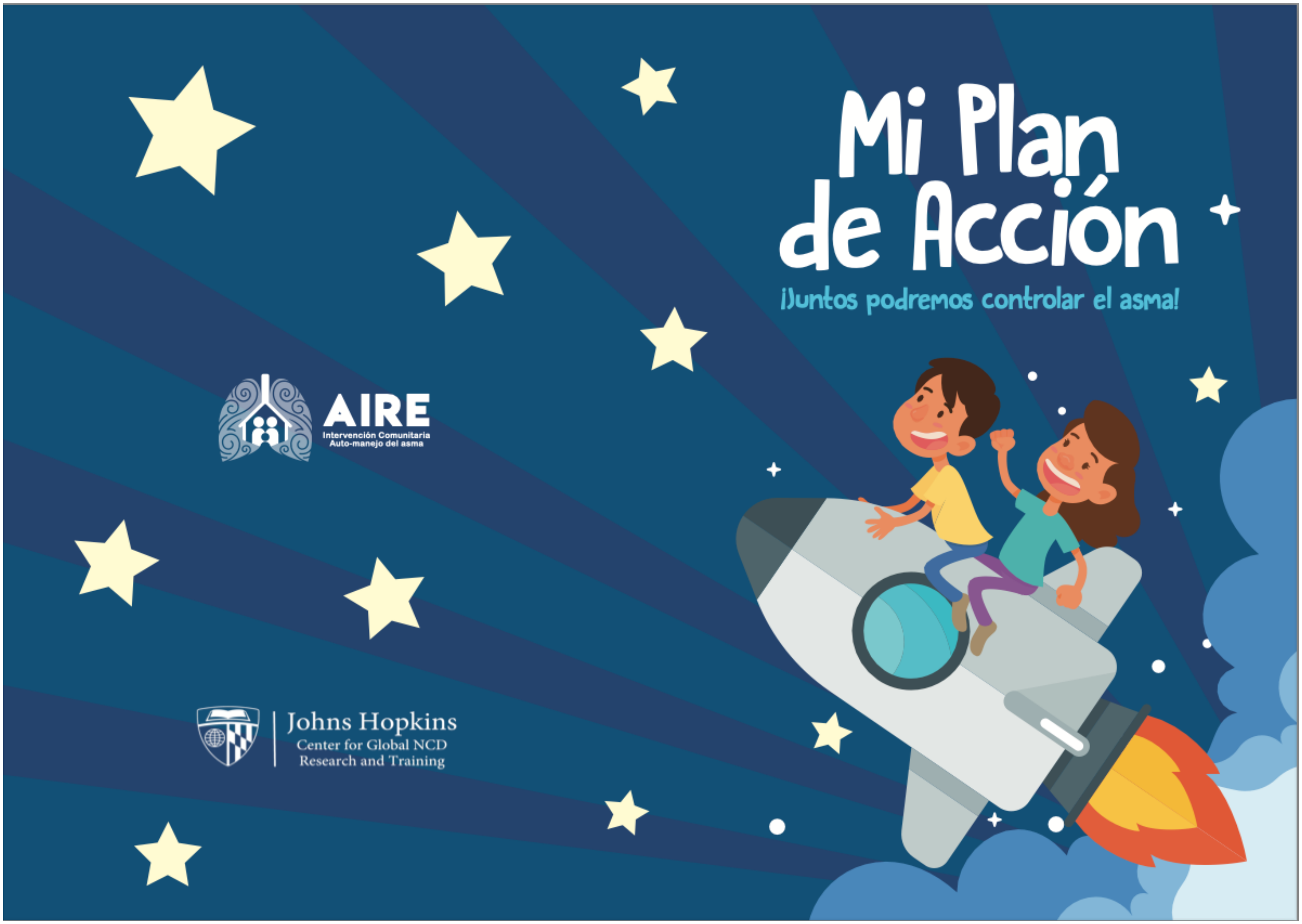
Mi Plan de Acción
¡Juntos podremos controlar el asma!
AIRE
Intervención Comunitaria
Auto-manejo del asma
Johns Hopkins
Center for Global NCD
Research and Training

Plan de Acción

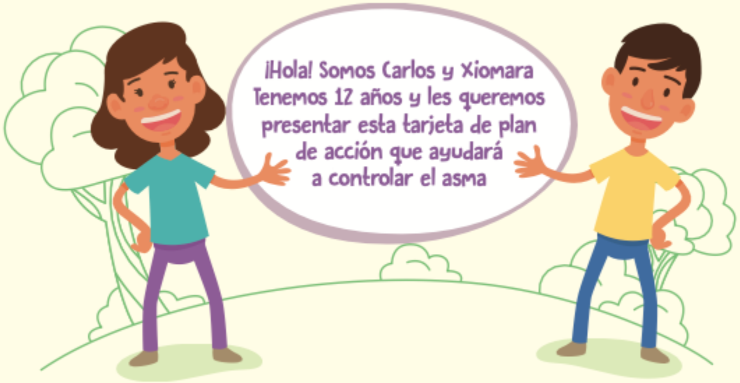

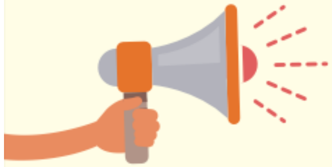

¿Qué es un plan de acción?

Es una herramienta que usa un sistema de zonas con colores del semáforo: verde, amarillo y rojo, que permite saber en qué zona está el asma y qué deben hacer cuando está en cada zona.

Nombre:

Teléfono:

Médico:

Diagnóstico:

Centro de Salud:

Teléf. de emergencia:

Enfermera monitora:

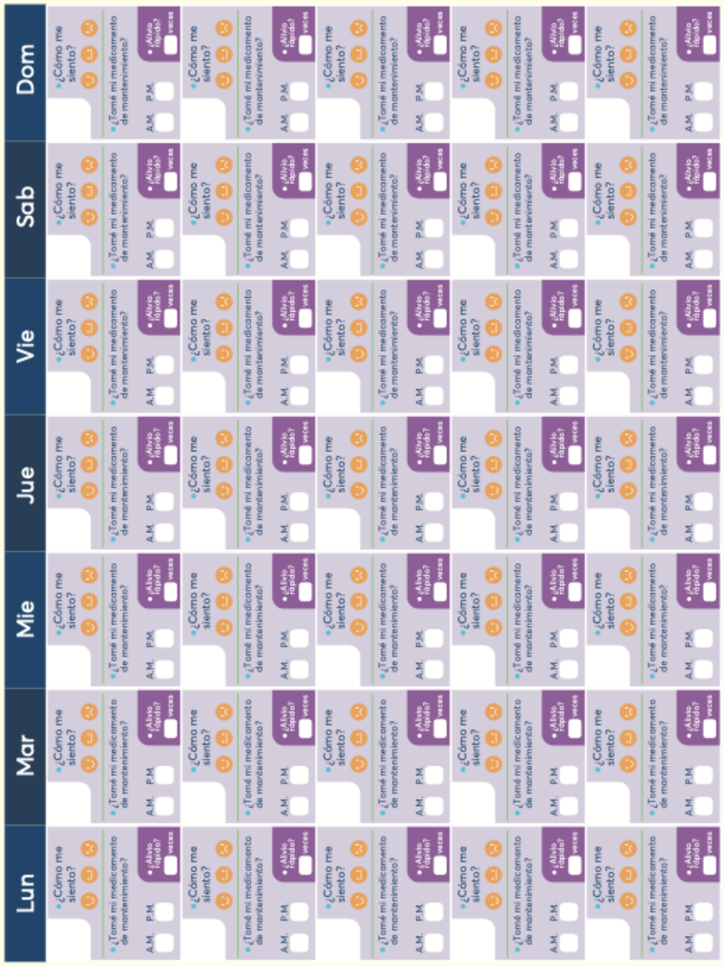

Día	Semana 1	Semana 2	Semana 3	Semana 4	Semana 5
Lun	¿Cómo me siento? / ¿Tomé mi medicamento de mantenimiento? A.M. P.M. / ¿Alivio rápido? veces	¿Cómo me siento? / ¿Tomé mi medicamento de mantenimiento? A.M. P.M. / ¿Alivio rápido? veces	¿Cómo me siento? / ¿Tomé mi medicamento de mantenimiento? A.M. P.M. / ¿Alivio rápido? veces	¿Cómo me siento? / ¿Tomé mi medicamento de mantenimiento? A.M. P.M. / ¿Alivio rápido? veces	¿Cómo me siento? / ¿Tomé mi medicamento de mantenimiento? A.M. P.M. / ¿Alivio rápido? veces
Mar	¿Cómo me siento? / ¿Tomé mi medicamento de mantenimiento? A.M. P.M. / ¿Alivio rápido? veces	¿Cómo me siento? / ¿Tomé mi medicamento de mantenimiento? A.M. P.M. / ¿Alivio rápido? veces	¿Cómo me siento? / ¿Tomé mi medicamento de mantenimiento? A.M. P.M. / ¿Alivio rápido? veces	¿Cómo me siento? / ¿Tomé mi medicamento de mantenimiento? A.M. P.M. / ¿Alivio rápido? veces	¿Cómo me siento? / ¿Tomé mi medicamento de mantenimiento? A.M. P.M. / ¿Alivio rápido? veces
Mie	¿Cómo me siento? / ¿Tomé mi medicamento de mantenimiento? A.M. P.M. / ¿Alivio rápido? veces	¿Cómo me siento? / ¿Tomé mi medicamento de mantenimiento? A.M. P.M. / ¿Alivio rápido? veces	¿Cómo me siento? / ¿Tomé mi medicamento de mantenimiento? A.M. P.M. / ¿Alivio rápido? veces	¿Cómo me siento? / ¿Tomé mi medicamento de mantenimiento? A.M. P.M. / ¿Alivio rápido? veces	¿Cómo me siento? / ¿Tomé mi medicamento de mantenimiento? A.M. P.M. / ¿Alivio rápido? veces
Jue	¿Cómo me siento? / ¿Tomé mi medicamento de mantenimiento? A.M. P.M. / ¿Alivio rápido? veces	¿Cómo me siento? / ¿Tomé mi medicamento de mantenimiento? A.M. P.M. / ¿Alivio rápido? veces	¿Cómo me siento? / ¿Tomé mi medicamento de mantenimiento? A.M. P.M. / ¿Alivio rápido? veces	¿Cómo me siento? / ¿Tomé mi medicamento de mantenimiento? A.M. P.M. / ¿Alivio rápido? veces	¿Cómo me siento? / ¿Tomé mi medicamento de mantenimiento? A.M. P.M. / ¿Alivio rápido? veces
Vie	¿Cómo me siento? / ¿Tomé mi medicamento de mantenimiento? A.M. P.M. / ¿Alivio rápido? veces	¿Cómo me siento? / ¿Tomé mi medicamento de mantenimiento? A.M. P.M. / ¿Alivio rápido? veces	¿Cómo me siento? / ¿Tomé mi medicamento de mantenimiento? A.M. P.M. / ¿Alivio rápido? veces	¿Cómo me siento? / ¿Tomé mi medicamento de mantenimiento? A.M. P.M. / ¿Alivio rápido? veces	¿Cómo me siento? / ¿Tomé mi medicamento de mantenimiento? A.M. P.M. / ¿Alivio rápido? veces
Sab	¿Cómo me siento? / ¿Tomé mi medicamento de mantenimiento? A.M. P.M. / ¿Alivio rápido? veces	¿Cómo me siento? / ¿Tomé mi medicamento de mantenimiento? A.M. P.M. / ¿Alivio rápido? veces	¿Cómo me siento? / ¿Tomé mi medicamento de mantenimiento? A.M. P.M. / ¿Alivio rápido? veces	¿Cómo me siento? / ¿Tomé mi medicamento de mantenimiento? A.M. P.M. / ¿Alivio rápido? veces	¿Cómo me siento? / ¿Tomé mi medicamento de mantenimiento? A.M. P.M. / ¿Alivio rápido? veces
Dom	¿Cómo me siento? / ¿Tomé mi medicamento de mantenimiento? A.M. P.M. / ¿Alivio rápido? veces	¿Cómo me siento? / ¿Tomé mi medicamento de mantenimiento? A.M. P.M. / ¿Alivio rápido? veces	¿Cómo me siento? / ¿Tomé mi medicamento de mantenimiento? A.M. P.M. / ¿Alivio rápido? veces	¿Cómo me siento? / ¿Tomé mi medicamento de mantenimiento? A.M. P.M. / ¿Alivio rápido? veces	¿Cómo me siento? / ¿Tomé mi medicamento de mantenimiento? A.M. P.M. / ¿Alivio rápido? veces

Semáforo de Control de asma

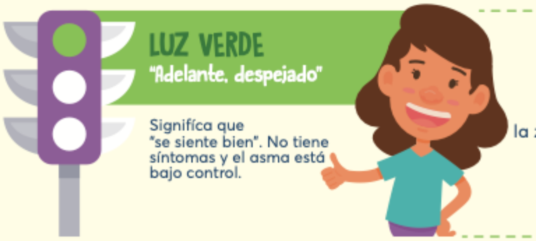

LUZ VERDE

"Adelante, despejado"

Significa que "se siente bien". No tiene síntomas y el asma está bajo control.

Cuando se está en la zona verde, el tratamiento está funcionando

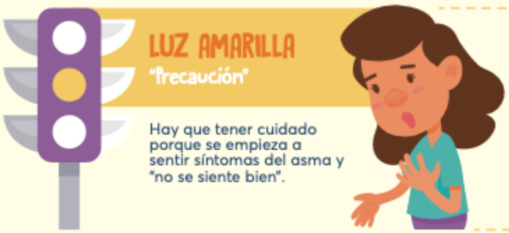

LUZ AMARILLA

"Precaución"

Hay que tener cuidado porque se empieza a sentir síntomas del asma y "no se siente bien".

Para saber que hacer, hay que seguir el plan de acción para el control del asma que corresponde a esta zona.

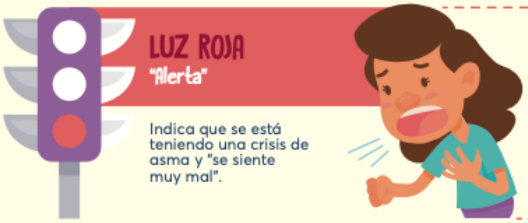

LUZ ROJA

"Alerta"

Indica que se está teniendo una crisis de asma y "se siente muy mal".

Esta zona es peligrosa, hay que buscar ayuda de inmediato. Seguir el plan de acción correspondiente a esta zona.

Plan de acción para el control del asma en casa

zona verde

SE SIENTE BIEN

- Sin tos.
- Sin silbidos al respirar (sibilancias).
- Sin opresión en el pecho, ni dificultad para respirar durante el día o la noche.
- Puede realizar sus actividades normales.

ANTES DE HACER EJERCICIO:

zona amarilla

SU ASMA ESTÁ EMPEORANDO

- Tiene tos.
- Tiene sibilancias.
- Tiene opresión en el pecho o dificultad para respirar.
- Se despierta de noche por el asma.
- Puede hacer algunas de sus actividades normales, pero no todas.

zona roja

¡ALERTA MÉDICA!

- Tiene mucha dificultad para respirar.
- Los medicamentos de alivio rápido no le han ayudado.
- No puede hacer sus actividades normales.
- Los síntomas son iguales o empeoran después de haber pasado 24 horas en la ZONA AMARILLA.

señales de peligro

- Tiene dificultad para caminar y hablar por la falta de aire.
- Tiene los labios o las uñas azules.

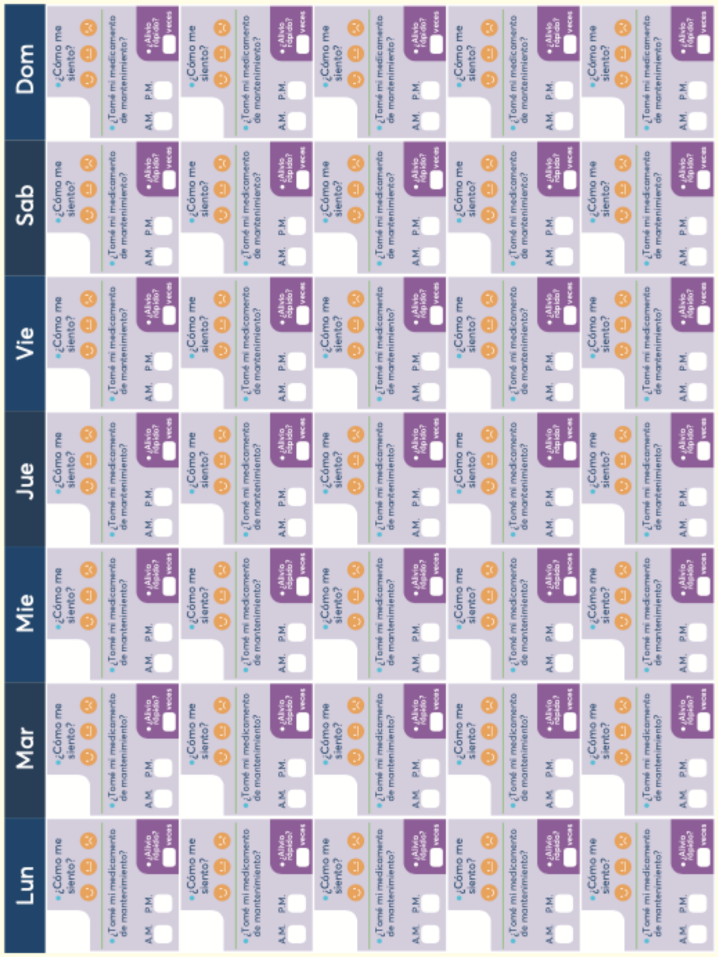

Lun	Mar	Mie	Jue	Vie	Sab	Dom
¿Cómo me siento? ¿Tomé mi medicamento de mantenimiento? A.M. P.M. ¿Alivio rápido? veces	¿Cómo me siento? ¿Tomé mi medicamento de mantenimiento? A.M. P.M. ¿Alivio rápido? veces	¿Cómo me siento? ¿Tomé mi medicamento de mantenimiento? A.M. P.M. ¿Alivio rápido? veces	¿Cómo me siento? ¿Tomé mi medicamento de mantenimiento? A.M. P.M. ¿Alivio rápido? veces	¿Cómo me siento? ¿Tomé mi medicamento de mantenimiento? A.M. P.M. ¿Alivio rápido? veces	¿Cómo me siento? ¿Tomé mi medicamento de mantenimiento? A.M. P.M. ¿Alivio rápido? veces	¿Cómo me siento? ¿Tomé mi medicamento de mantenimiento? A.M. P.M. ¿Alivio rápido? veces
¿Cómo me siento? ¿Tomé mi medicamento de mantenimiento? A.M. P.M. ¿Alivio rápido? veces	¿Cómo me siento? ¿Tomé mi medicamento de mantenimiento? A.M. P.M. ¿Alivio rápido? veces	¿Cómo me siento? ¿Tomé mi medicamento de mantenimiento? A.M. P.M. ¿Alivio rápido? veces	¿Cómo me siento? ¿Tomé mi medicamento de mantenimiento? A.M. P.M. ¿Alivio rápido? veces	¿Cómo me siento? ¿Tomé mi medicamento de mantenimiento? A.M. P.M. ¿Alivio rápido? veces	¿Cómo me siento? ¿Tomé mi medicamento de mantenimiento? A.M. P.M. ¿Alivio rápido? veces	¿Cómo me siento? ¿Tomé mi medicamento de mantenimiento? A.M. P.M. ¿Alivio rápido? veces
¿Cómo me siento? ¿Tomé mi medicamento de mantenimiento? A.M. P.M. ¿Alivio rápido? veces	¿Cómo me siento? ¿Tomé mi medicamento de mantenimiento? A.M. P.M. ¿Alivio rápido? veces	¿Cómo me siento? ¿Tomé mi medicamento de mantenimiento? A.M. P.M. ¿Alivio rápido? veces	¿Cómo me siento? ¿Tomé mi medicamento de mantenimiento? A.M. P.M. ¿Alivio rápido? veces	¿Cómo me siento? ¿Tomé mi medicamento de mantenimiento? A.M. P.M. ¿Alivio rápido? veces	¿Cómo me siento? ¿Tomé mi medicamento de mantenimiento? A.M. P.M. ¿Alivio rápido? veces	¿Cómo me siento? ¿Tomé mi medicamento de mantenimiento? A.M. P.M. ¿Alivio rápido? veces
¿Cómo me siento? ¿Tomé mi medicamento de mantenimiento? A.M. P.M. ¿Alivio rápido? veces	¿Cómo me siento? ¿Tomé mi medicamento de mantenimiento? A.M. P.M. ¿Alivio rápido? veces	¿Cómo me siento? ¿Tomé mi medicamento de mantenimiento? A.M. P.M. ¿Alivio rápido? veces	¿Cómo me siento? ¿Tomé mi medicamento de mantenimiento? A.M. P.M. ¿Alivio rápido? veces	¿Cómo me siento? ¿Tomé mi medicamento de mantenimiento? A.M. P.M. ¿Alivio rápido? veces	¿Cómo me siento? ¿Tomé mi medicamento de mantenimiento? A.M. P.M. ¿Alivio rápido? veces	¿Cómo me siento? ¿Tomé mi medicamento de mantenimiento? A.M. P.M. ¿Alivio rápido? veces
¿Cómo me siento? ¿Tomé mi medicamento de mantenimiento? A.M. P.M. ¿Alivio rápido? veces	¿Cómo me siento? ¿Tomé mi medicamento de mantenimiento? A.M. P.M. ¿Alivio rápido? veces	¿Cómo me siento? ¿Tomé mi medicamento de mantenimiento? A.M. P.M. ¿Alivio rápido? veces	¿Cómo me siento? ¿Tomé mi medicamento de mantenimiento? A.M. P.M. ¿Alivio rápido? veces	¿Cómo me siento? ¿Tomé mi medicamento de mantenimiento? A.M. P.M. ¿Alivio rápido? veces	¿Cómo me siento? ¿Tomé mi medicamento de mantenimiento? A.M. P.M. ¿Alivio rápido? veces	¿Cómo me siento? ¿Tomé mi medicamento de mantenimiento? A.M. P.M. ¿Alivio rápido? veces

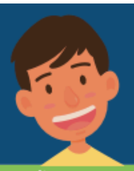

Tome estos medicamentos de control a largo plazo todos los días (incluído antiinflamatorio)

Medicamento	Cuándo debe tomar	Cúanto debe tomarlo

______ ◯ 2 o ◯ 4 descargas ______ 5 minutos de hacer ejercicio

Agregue el medicamento de alivio rápido y siga tomando el medicamento de la ZONA VERDE

- ______ ◯ 2 o ◯ 4 descargas cada 20 minutos por un máximo de 1 hora.
  Si sus síntomas regresan a la ZONA VERDE después de 1 hora del tratamiento anterior.
- ◯ Continúe vigilándolos para asegurarse de permanecer en la ZONA VERDE.
  Si no regresa, después de una hora a la ZONA VERDE, acudir al médico más cercano o acudir a su médico tratante.

Tome este medicamento:

- ______ ◯ 2 o ◯ 6 descargas.
- ◯ ______mg. Luego, llame al doctor INMEDIATAMENTE.
- △ No se ha podido comunicar con el doctor.
- △ Todavía está en la ZONA ROJA después de 15 minutos vaya al hospital más cercano, "Sergio Bernales", "Cayetano Heredia" o a la Red asistencial de EsSalud donde pertenezca.

- Tome ◯ 4 ◯ 6 descargas del medicamento de alivio rápido y ______
- Vaya al hospital o llame al ______

¡COMO SER UNOS SUPERNIÑ@S CON ASMA!

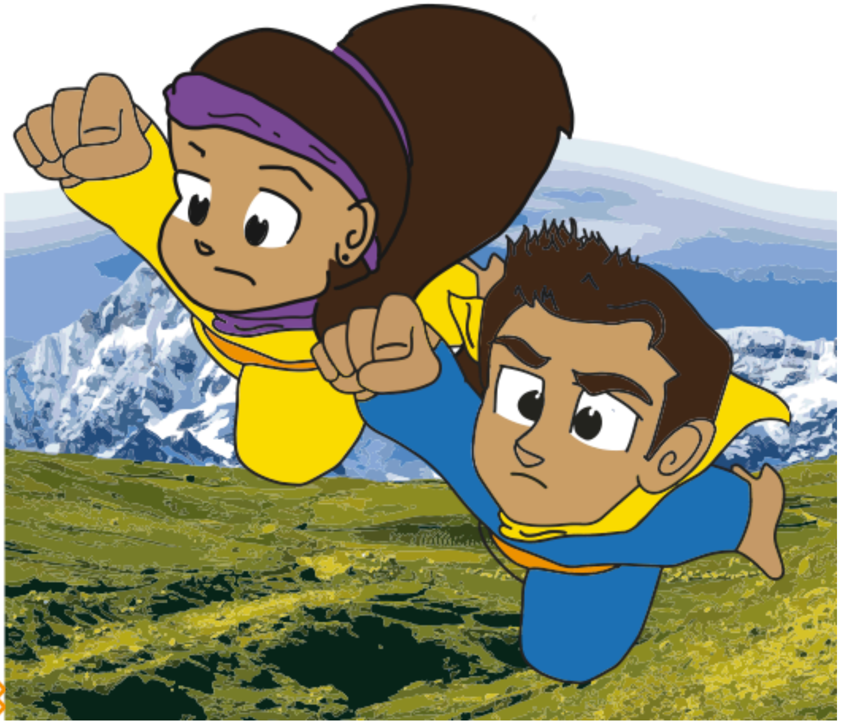

SI TE RECUERDAS DE LA PALABRA "*INCA*", PODRÁS RECORDAR LAS CUATRO PLABRAS CLAVES:

- **I**NHALADOR
- **N**ÚMEROS DE EMERGENCIA
- **C**ONOCIMIENTO
- PLAN DE **A**CCIÓN

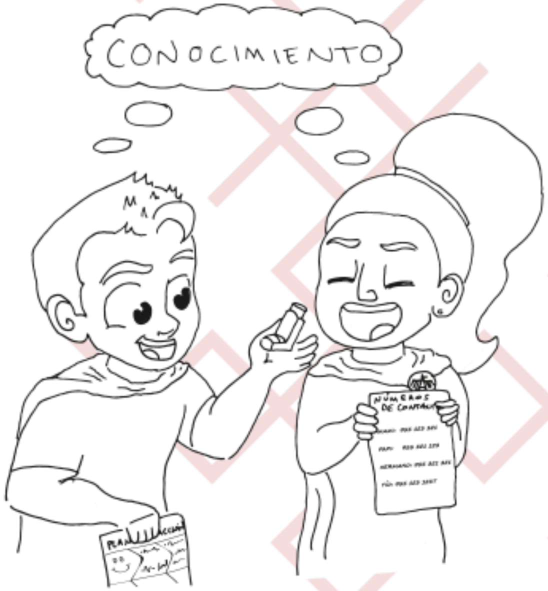

ESTIMADOS PADRES,

ESTE MANUAL FUE DISEÑADO PARA AYUDAR A QUE SUS HIJOS/AS RECUERDEN LAS COSAS BÁSICAS DEL CONTROL DE SU ASMA. POR FAVOR AYUDE A SUS HIJOS/AS EN COMPLETAR Y ENTENDER LOS CONTENIDOS DE ESTE MANUAL PARA QUE SEA LO MAS ÚTIL POSIBLE PARA ELLOS. USE EL MANUAL COMO UN GUÍA, Y SIÉNTASE LIBRE DE HABLAR CON SU HIJO/A SOBRE EL ASMA Y SOBRE SU CONDICIÓN EN PARTICULAR.

-PRISMA

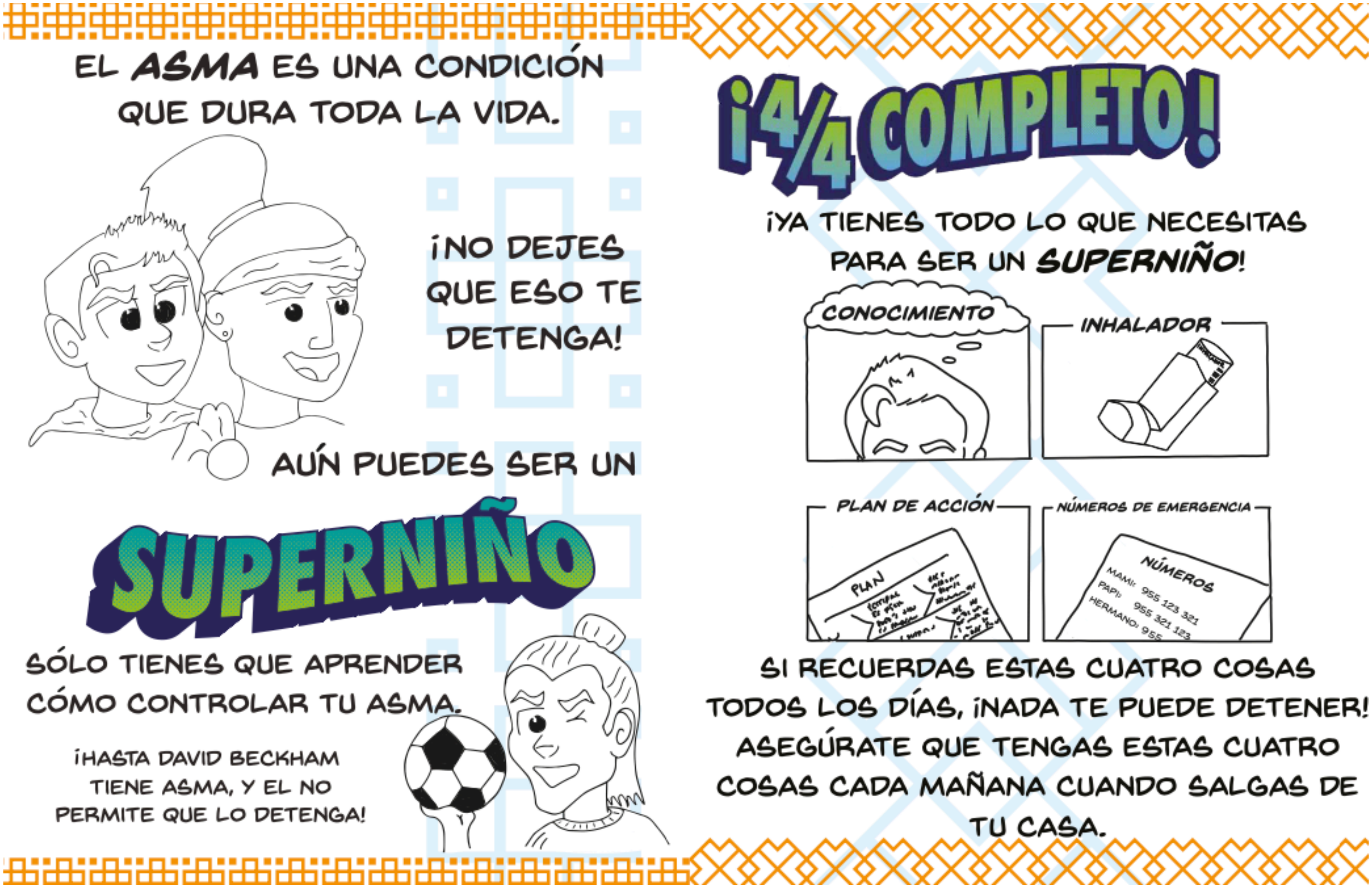
EL ASMA ES UNA CONDICIÓN QUE DURA TODA LA VIDA.
¡NO DEJES QUE ESO TE DETENGA!
AÚN PUEDES SER UN
SUPERNIÑO
SÓLO TIENES QUE APRENDER CÓMO CONTROLAR TU ASMA.
¡HASTA DAVID BECKHAM TIENE ASMA, Y EL NO PERMITE QUE LO DETENGA!
¡4/4 COMPLETO!
¡YA TIENES TODO LO QUE NECESITAS PARA SER UN SUPERNIÑO!
CONOCIMIENTO
INHALADOR
PLAN DE ACCIÓN
PLAN
NÚMEROS DE EMERGENCIA
NÚMEROS
MAMI: 955 123 321
PAPI: 955 321 123
SI RECUERDAS ESTAS CUATRO COSAS TODOS LOS DÍAS, ¡NADA TE PUEDE DETENER!
ASEGÚRATE QUE TENGAS ESTAS CUATRO COSAS CADA MAÑANA CUANDO SALGAS DE TU CASA.
